# Targeting SIRT3 sensitizes glioblastoma to ferroptosis by promoting mitophagy and inhibiting SLC7A11

**DOI:** 10.1038/s41419-024-06558-0

**Published:** 2024-02-23

**Authors:** Xiaohe Li, Wenlong Zhang, Zhengcao Xing, Shuming Hu, Geqiang Zhang, Tiange Wang, Tianshi Wang, Qiuju Fan, Guoqiang Chen, Jinke Cheng, Xianguo Jiang, Rong Cai

**Affiliations:** 1 Department of Biochemistry & Molecular Cell Biology, Key Laboratory of Cell Differentiation and Apoptosis of Chinese Ministry of Education, Shanghai Jiao Tong University School of Medicine, Shanghai, 200025 China; 2 Shanghai Immune Therapy Institute, Renji Hospital, Shanghai Jiao Tong University School of Medicine, Shanghai, 200025 China; 3 State Key Laboratory of Oncogenes and Related Genes, Renji Hospital Affiliated, Shanghai Key Laboratory for Tumor Microenvironment and Inflammation, Department of Biochemistry & Molecular Cell Biology, Shanghai Jiao Tong University School of Medicine, Shanghai, 200025 China; 4 Department of Neurology, Renji Hospital, Shanghai Jiao Tong University School of Medicine, Shanghai, 200025 China

**Keywords:** Cell death, Cancer

## Abstract

Glioblastoma (GBM) cells require large amounts of iron for tumor growth and progression, which makes these cells vulnerable to destruction *via* ferroptosis induction. Mitochondria are critical for iron metabolism and ferroptosis. Sirtuin-3 (SIRT3) is a deacetylase found in mitochondria that regulates mitochondrial quality and function. This study aimed to characterize SIRT3 expression and activity in GBM and investigate the potential therapeutic effects of targeting SIRT3 while also inducing ferroptosis in these cells. We first found that SIRT3 expression was higher in GBM tissues than in normal brain tissues and that SIRT3 protein expression was upregulated during RAS-selective lethal 3 (RSL3)-induced GBM cell ferroptosis. We then observed that inhibition of SIRT3 expression and activity in GBM cells sensitized GBM cells to RSL3-induced ferroptosis both in vitro and in vivo. Mechanistically, SIRT3 inhibition led to ferrous iron and ROS accumulation in the mitochondria, which triggered mitophagy. RNA-Sequencing analysis revealed that upon SIRT3 knockdown in GBM cells, the mitophagy pathway was upregulated and SLC7A11, a critical antagonist of ferroptosis *via* cellular import of cystine for glutathione (GSH) synthesis, was downregulated. Forced expression of SLC7A11 in GBM cells with SIRT3 knockdown restored cellular cystine uptake and consequently the cellular GSH level, thereby partially rescuing cell viability upon RSL3 treatment. Furthermore, in GBM cells, SIRT3 regulated *SLC7A11* transcription through ATF4. Overall, our study results elucidated novel mechanisms underlying the ability of SIRT3 to protect GBM from ferroptosis and provided insight into a potential combinatorial approach of targeting SIRT3 and inducing ferroptosis for GBM treatment.

## Introduction

Glioblastoma (GBM) is a highly malignant tumor with a poor prognosis, and research has established that GBM cells have a high demand on iron during tumor growth and invasion [1]. As one of the most abundant elements in the human body, iron plays essential roles in many biological processes, including oxygen transport, heme biosynthesis, DNA replication, electron transport, *etc*. In comparison with normal cells, GBM cells exhibit abnormal expression of iron-metabolism related proteins and altered levels of iron-related enzyme activities, which provide high availability of iron in GBM cells [1]. This high availability of iron allows GBM growth and progression but also makes GBM cells more susceptible than normal cells to ferroptosis [2].

Ferroptosis is a form of regulated cell death (RCD) described by Stockwell and colleagues about a decade ago [3]. Ferroptosis is an iron-dependent cell death driven by the accumulation of lipid peroxides (LPOs), which makes it distinct from apoptosis and other forms of cell death [4]. In recent years, ferroptosis has increasingly emerged as an important process in the progression of multiple cancers, including GBM [5–7]. Understanding how ferroptosis is triggered specifically in GBM cells could provide insight for the development of an approach to treat GBM effectively in the clinic while leaving normal brain cells unaffected.

In 2019, mitochondria were shown to play a crucial role in ferroptosis induced by cysteine deprivation [8]. Mitochondria-dependent ferroptosis also was shown to play a pivotal role in doxorubinxin-induced cardiotoxicity [9]. The mitochondria of ferroptotic cells display typical morphology changes, including a shrunken appearance and loss of cristae, indicating that mitochondrial dysfunction is involved in ferroptosis. ZZW-115, an inhibitor of the stress-inducible protein nuclear protein 1 (NUPR1), was found induce tumor cell ferroptosis in a mitochondria-dependent manner [10].

SIRT3 (sirtuin-3) is a deacetylase that is located and functions primarily in mitochondria [11]. SIRT3 acts as either an oncogene or tumor suppressor by regulating cell death and growth [12, 13]. Research on the role of SIRT3 in tumor cell ferroptosis is only emerging, and the results so far have been inconsistent. In gallbladder cancer, SIRT3 was found to promote ferroptosis by activating the AKT signaling pathway [14], but in another study, SIRT3 was shown to have an inhibitory role in ferroptosis in human cancer cells in a p53-dependent manner [15]. The function of SIRT3 in ferroptosis of GBM cells remains completely unknown. Accordingly, the present study investigated the roles and mechanisms of SIRT3 in GBM ferroptosis and progression.

## Materials and methods

### Antibodies and reagents

The antibodies and reagents used in this study are described in Table S1.

### Bioinformatics analysis

The UALCAN (https://ualcan.path.uab.edu/) online tool was used to analyze the expression of SIRT3 in GBM tissues and normal brain tissues. Correlation between SIRT3 and SLC7A11 expression levels was analyzed by calculating the Spearman’s rank correlation coefficient.

### Tissue microarray

A combined tissue microarray of GBM and normal brain tissues was purchased from Xi’an Taibs Pharmaceutical Technology Co., Ltd., with pathological grade reagents (Table S2), and stored at 4° C. The correlation between SIRT3 and SLC7A11 was analyzed through Spearman’s rank correlation coefficient.

### Cell lines and culture

Two GBM cell lines, U251 and U87MG, were purchased from NANJING COBIOER BIOSCIENCES CO. LTD. U251 cells were cultured in Dulbecco’s modified Eagle’s medium (DMEM)-high glucose with 10% fetal bovine serum (FBS) and 1% penicillin-streptomycin solution (100×) at 37 °C in a humidified atmosphere of 5% CO_2_. U87MG cells were cultured in minimum essential medium (MEM) with 10% FBS, 1% penicillin-streptomycin solution (100×), 1% MEM non-essential amino acids solution (100×) and 1% sodium pyruvate (100 mM) at 37 °C in a humidified atmosphere of 5% CO_2_.

### Erastin and RSL3 treatment

According to the results of the concentration gradient test, we treated U251 or U87MG cells with erastin or RAS-selective lethal 3 (RSL3) for 24 h.

### Cell viability assay

U251 cells or U87MG cells were seeded in 96-well plates (6 × 10^3^ cells/well) and cultured for 12 h at 37 °C in a humidified atmosphere of 5% CO_2_. Next, cells were treated with erastin (0, 5, 10, or 20 μM) or RSL3 (0, 1, 2, 4, 6, or 8 μM) at 37 °C for 24 h in a humidified atmosphere of 5% CO_2_. Then, cells were exposed to 100 μL culture medium with 10 μL Cell Counting Kit-8 (CCK8) solution for 1 h at 37 °C in a 5% CO_2_ incubator. Subsequently, the 96-well plate was removed from the incubator, and the absorbance of the solution in each well determined by a microplate reader (450 nm). Cell viability was then calculated.

### Transmission electron microscopy

Cells were seeded in 10-cm dishes and cultured for 24 h at 37 °C in a 5% CO_2_ incubator. After removal of the supernatant, 8 mL fixative was added to each dish for fixation at room temperature for 2–4 h. Then the cells were scraped and collected into a 15-mL tube for centrifugation at 800 rpm for 5 min. The resulting cell pellets were fixed in 2% osmium tetroxide and dehydrated in gradual series of ethanol and propylene oxide. Next the cell pellets were embedded in Epon and stored at 60 °C for 20 h. Ultrathin sections were placed onto 200 mesh copper grids and double-stained with lead citrate and uranyl acetate. Finally, the ultrathin sections were observed with transmission electron microscopy.

### Western blotting

For western blotting, cell cultures were washed twice with phosphate-buffered saline (PBS) before the addition of sodium dodecyl sulfate (SDS) lysis buffer with 1% phenylmethanesulfonyl fluoride (PMSF) and 1% protease inhibitor cocktail. After the cells were fully lysed, the protein lysates were transferred to a 1.5-mL Eppendorf (EP) tube and sonicated three times for 5 s each time. The lysate solutions were then heated at 100 °C for 10 min before centrifugation at 13,000 rpm for 10 min. The supernatant was transferred to a new 1.5-mL EP tube, and the protein concentration was measured via NanoDrop analysis. The protein concentration in all samples was uniformly adjusted to 1.25 μg/μL by adding 5× Loading Buffer. The samples were then heated at 100 °C for 10 min before separation of the proteins by SDS-polyacrylamide gel electrophoresis (PAGE).

### RNA isolation and quantitative reverse transcriptase-polymerase chain reaction (qRT-PCR)

Cells were seeded on 6-well plates and cultured for 24 h. Then, cells were washed twice with PBS before being lysed in lysis buffer. RNA was extracted using TRNzol Universal Reagent (TIANGEN) according to the manufacturer’s protocol. cDNA was obtained by reverse transcription of 1 μg RNA using PrimeScript™ RT Master Mix (Takara). The primers used are listed in Table S3. qRT-PCR was performed using the ChamQ Universal SYBR qPCR Master Mix. Subsequently, samples (*n* = 3) were analyzed on a LightCycler480 Instrument (Roche).

### DFO and NAC treatment

U251 cells or U87MG cells were seeded in 96-well plates (6 × 10^3^ cells/well) for culture and treated with medium containing dimethyl sulfoxide (DMSO), deferoxamine (DFO, 10 μM) or N-acetylcysteine (NAC, 0.5 mM) for 12 h at 37 °C in a 5% CO_2_ incubator. Next cells were treated with RSL3 (6 μM) for 24 h at 37 °C in a 5% CO_2_ incubator. Further analyses were carried out as described elsewhere in this section.

### Lipid peroxidation assay

U251 cells were treated with or without RSL3 for 4 h. Then cells were digested from culture dishes, collected into a 1.5-mL tube, and centrifuged at 800 rpm at room temperature for 5 min. The cells were then washed twice with 1 mL PBS and suspended in 200 μL PBS before transfer to a round-bottom 96-well plate. Next the cells were centrifuged at 800 rpm for 5 min at room temperature and suspended in 200 μL BODIPY (5 μM) for treatment for 30 min at 37 °C in a 5% CO_2_ incubator. Subsequently, the cells were washed twice with 200 μL PBS and suspended in 200 μL PBS. The lipid peroxidation levels were detected by flow cytometry (excitation: 665 nm, emission: 676 nm).

### GBM#4 isolation

We isolated GBM#4 cells from primary surgical GBM biopsy specimens from patients treated at Renji Hospital Affiliated to Shanghai Jiao Tong University School of Medicine in accordance with the study protocol. The study was explained to the participants, and informed consent was obtained. GBM cells were cultured in DMEM supplemented with 10% FBS and 1% penicillin-streptomycin solution.

### shRNA and stable cell lines

The plasmids used in this study were PLKO.1-Puro, PLKO.1-shSIRT3-2-Puro, PLKO.1-shSIRT3-4-Puro, PGMLV-CMV-MCS-PGK, PGMLV-CMV-H_SLC7A11-PGK, PGMLV-CMV-H_ACO1(IRP1)-HA-PGK, PGMLV-CMV-MCS-3×Flag-EF1-ZsGreen1-T2A-Blasticidin, and PGMLV-CMV-H_ATF4-3×Flag-EF1-ZsGreen1-T2A-Blasticidin. Lentiviruses were packaged with plasmids PSPA×2 and PMD.2 G and used to infect U251, U87MG and GBM#4 cells. Infected cells were selected with puromycin/blasticidin-containing media for more than 3 days to establish stable cell sublines.

### 3-TYP treatment

U251 or GBM#4 cells were seeded in 96-well plates (6 × 10^3^ cells/well) and treated with DMSO or 3-TYP (10 or 20 μM) for 12 h at 37 °C in a 5% CO_2_ incubator. Further analyses were as described elsewhere in this section.

### Mitochondrial acetylation assay

Cells were collected into a 1.5-μL EP tube and centrifuged at 370 g for 10 min at 4 °C. After removal of the supernatant, the cells were resuspended in 500 μL NKM buffer and centrifuged again at 370 g for 10 min at 4 °C. The cells were resuspended in 250 μL homogenization buffer containing 1% PMSF and 1% dithiothreitol (DTT) and placed in an ice bath for 10 min. Then, a mitochondrial grinder was applied 35 times before addition of an equal volume of 2 M sucrose buffer. The solution was centrifuged at 1200 g for 5 min at 4 °C, and then the supernatant was transferred to a new 1.5-mL EP tube. This process was repeated three times. The solution was then centrifuged at 7000 g for 10 min at 4 °C, and after removal of the supernatant, the pellet was resuspended in 200 μL mitochondrial suspension buffer containing 1% PMSF and 1% DTT before centrifugation at 9500 g for 5 min at 4 °C. Upon removal of the supernatant, this centrifugation step was repeated twice to obtain the mitochondrial pellets. Western Blotting was used to detect the mitochondrial acetylation level.

### Animal experiments

All animal experiments in this study were approved by the Animal Care Committee of Shanghai Jiao Tong University School of Medicine (Shanghai, China), and all experiments were performed following the Guidelines for the Care and Use of Laboratory Animals issued by the Chinese Council on Animal Research. Four-week-old nude mice (male BALB/c nu/nu athymic nude mice) were adapted to the environment for 1 week. Mice were randomly divided into 3 groups, and U251 cells (10^7^/sample) were suspended in 100 μL of a mixture of PBS and Matrigel (1:1) and injected in the armpits of 5 nude mice in each group. Tumor size was measured every 3 days from 1 week after the injection. Once the tumor volume exceeded 10 mm^3^, 3-TYP (50 mg/kg) and RSL3 (50 mg/kg) were injected into the tumor every 2 days alternatively for 8 days. After that, the mice were sacrificed.

### Detection of malondialdehyde (MDA) level

GBM cells were digested from the culture dish, collected into a 1.5-mL tube, and centrifuged at 800 rpm for 5 min. Cells were washed once with 1 mL PBS and suspended in 100 μL antioxidant PBS solution. After the cells in each treatment group were completely lysed with 100 μL lysis buffer, each mixture was mixed thoroughly in 250 μL working solution. Then cells were heated at 95 °C for 15 min. After cooling, the mixture was centrifuged at 10,000 × *g* for 10 min, and the supernatant was collected. Then the MDA levels were detected by a fluorescence microplate reader (Ex: 540 nm, Em: 590 nm). For tumor tissue, homogenate was lysed in PBS with a ratio of tissue to reagent of 1:10. After lysis of the homogenate, samples were centrifuged at 10,000 g for 10 min at 4 °C, and the supernatant was collected for subsequent MDA detection according to the protocol for the DOJINDO MDA Assay Kit.

### Measurement of GSH and GSSG levels

Tissues were quickly frozen with liquid nitrogen and then ground into a powder. For every 10 mg of ground tissue powder, 30 μL of Protein Removal Reagent Solution was added, and the mixture was fully vortexed. Then another 70 μL of Protein Removal Reagent Solution was added, and the tissue powder was fully homogenized with a glass homogenizer. After standing at 4 °C for 10 min, each sample was centrifuged at 10,000 g at 4 °C for 10 min, and the supernatant was collected to determine total glutathione levels. Glutathione (GSH) and oxidized glutathione (GSSG) levels were detected according to the protocol for the Beyotime Assay Kit GSH and GSSG Assay Kit.

### Immunohistochemistry

Harvested tumor xenografts were embedded in paraffin following a routine procedure and sliced into 5-μm-thick tissue sections. After the tissue sections were deparaffinized and rehydrated in distilled water, they were placed in 3% hydrogen peroxide to block endogenous peroxidase activity. Then, the tissue sections were blocked in 3% bovine serum albumin (BSA) at room temperature for 30 min before being laid flat in a wet box and incubated overnight at 4 °C. Subsequently, the sections were washed three times with PBS and covered with secondary antibody (horseradish peroxidase [HRP] labeled) for 50 min at room temperature. The tissue sections were finally stained with 3, 3′-diaminobenzidine (DAB) solution and imaged under a microscope.

### Detection of mitochondrial Fe^2+^ level

Cells were seeded in a 3.5-cm glass-bottomed dish and cultured for 24 h at 37 °C in a 5% CO_2_ incubator. Then they were washed three times with 1 mL serum-free DMEM before being treated with 1 mL FerroOrange working solution (1 μmol/L) or Mito-FerroGreen working solution (5 μmol/L) freshly mixed with serum-free DMEM for 30 min at 37 °C in a 5% CO_2_ incubator. The cells were examined using a Leica TCS Sp8 STED Confocal Super-Resolution Microscope. After treatment with 1 mL FerroGreen working solution (5 μmol/L) for 30 min at 37 °C in a 5% CO_2_ incubator, the cells were washed twice with PBS and digested with Trypsin-EDTA solution (0.25%). The resulting supernatant was transferred to a 1.5-mL EP tube and then centrifuged at 800 rpm for 5 min. After removal of the supernatant, the cells were resuspended in 1 mL PBS and centrifuged again at 800 rpm for 5 min. Then cells were resuspended in 200 μL PBS and analyzed using a BD LSRFortessa™ Cell Analyzer, with a green (DCFH-DA) fluorescence channel.

### Cellular and mitochondrial ROS measurement

For the detection of reactive oxygen species (ROS) levels, cells were washed twice with PBS. After the supernatant was discarded, 1 mL dichlorodihydrofluorescein diacetate (DCFH-DA) working solution (1:1000) or MitoSOX working solution (1:1000) freshly mixed in serum-free DMEM was added into the wells and reacted for 20 min at 37 °C in a 5% CO_2_ incubator. Then the cells were washed twice with PBS and digested with Trypsin-EDTA solution (0.25%). The resulting supernatant was transferred to a 1.5-mL EP tube and then centrifuged at 800 rpm for 10 min. After removal of the supernatant, the cells were resuspended in 1 mL PBS and centrifuged again at 800 rpm for 5 min. This process was repeated three times after which the cells were resuspended in 200 μL PBS and analyzed using a Beckman CytoFlex S, with a green (DCFH-DA) or red (MitoSOX) fluorescence channel.

### RNA-Seq

Short hairpin negative control (shNC)-, shSIRT3#2- and shSIRT3#4-infected cells were seeded in 6-cm dishes and cultured for 24 h at 37 °C in a 5% CO_2_ incubator. Total RNA was isolated as described above and submitted to the Sequencing Diagnostic Platform of Shanghai Institute of Immunology, School of Medicine, Shanghai Jiaotong University (Shanghai, China) for RNA-Sequencing (RNA-Seq) analysis. Gene Set Enrichment Analysis (GSEA) and the Kyoto Encyclopedia of Genes and Genomes (KEGG) pathway and network analysis were performed on the results of RNA-Seq (significance defined as *p* < 0.05).

### Mdivi-1 treatment

U251 cells were seeded in 6-cm dishes and treated with DMSO, 3-TYP (20 μM), or Mdivi-1 (10 μM) for 12 h at 37 °C in a 5% CO_2_ incubator. Further analyses were conducted as described in other Materials and Methods subsections.

### Cystine uptake assay

For detection of cystine uptake, cells were seeded in 96-well plates and cultured for 24 h at 37 °C in a 5% CO_2_ incubator. The amount of cystine uptake was detected according to the protocol for the DOJINDO Cystine Uptake Assay Kit.

### Statistical analysis

All results were expressed as mean ± standard deviation (SD) for statistical analysis, which was performed using GraphPad Prism 9 and SPSS 25.0. Unpaired two-tailed Student’s *t*-test and two-way analysis of variance (ANOVA) were conducted to compare the means between or among different groups. Differences for which *p* < 0.05 were considered statistically significant.

## Results

### High expression of SIRT3 in GBM predicts poor prognosis

**Fig. 1:**
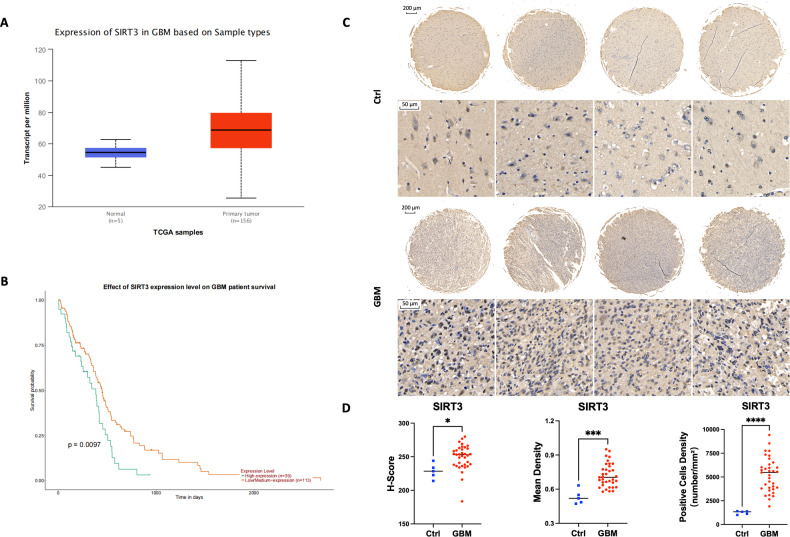
High expression of SIRT3 in GBM predicted poor prognosis. **A** UALCAN online analysis (https://ualcan.path.uab.edu/) revealed higher expression of SIRT3 in GBM tissues compared with normal brain tissues. **B** UALCAN online analysis revealed that high expression of SIRT3 in GBM predicts poor prognosis. **C** Representative results of tissue microarray assay showing that SIRT3 expression was higher in GBM tissues. **D** Quantitative results for SIRT3 expression in tissue microarray. *p < 0.05, **p < 0.01, ***p < 0.001, ****p < 0.0001.

To explore the role of SIRT3 in GBM, we first analyzed SIRT3 expression in GBM and normal brain tissue samples included in The Cancer Genome Atlas (TCGA) database. Our analysis showed that SIRT3 expression was higher in GBM tumor tissues than in normal tissues (Fig. 1A). Moreover, higher expression of SIRT3 predicted poor prognosis in GBM patients, as shown by UALCAN online analysis (Fig. 1B). Consistently, the results of a tissue microarray staining assay also showed that SIRT3 expression was higher in GBM tissues than in normal control brain samples (Fig. S1, Fig. 1C, D). These results collectively indicate that SIRT3 may be associated with GBM progression. We next used the Gene Expression Profile Interactive Analysis (GEPIA) database (http://gepia.cancer-pku.cn/) to analyze the correlation between SIRT3 expression and that of ferroptosis-related genes (FRGs) in GBM. We found a high positive correlation between SIRT3 and SLC7A11 (solute family 7 member 11) expression (Fig. S2). SLC7A11 is a cystine transporter essential for cellular GSH synthesis and thereby GPX4 (glutathione peroxidase 4) activity, and the observed correlation suggests that SIRT3 might promote GBM progression by antagonizing ferroptosis.

### SLC7A11 is highly expressed and positively correlated with SIRT3 expression in GBM tissues

**Fig. 2:**
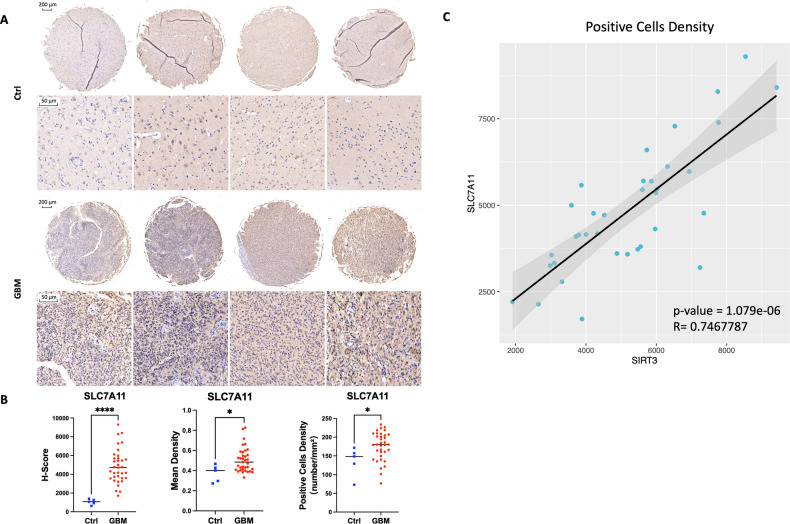
SLC7A11 expression was higher in GBM tissues than in normal brain tissues and highly positively correlated with SIRT3 expression. **A** Representative results of tissue microarray showing that SLC7A11 expression was higher in GBM patients. **B** Quantitative results of SLC7A11 expression from tissue microarray. *p < 0.05, ****p < 0.0001. **C** Correlation analysis revealed SIRT3 expression was highly correlated with SLC7A11 expression in GBM tissues.

To confirm the high positive correlation between SIRT3 and SLC7A11 expression, we also applied tissue microarray staining to compare the expression levels of SLC7A11 between GBM and normal brain tissues (Fig. S3). As shown in Fig. 2A, B, SLC7A11 expression was obviously higher in GBM tissues. Moreover, correlation analysis revealed a significant positive correlation between SIRT3 and SLC7A11 expression (Fig. 2C), indicating the potential protective role of SIRT3 against GBM cell ferroptosis.

### Erastin and RSL3 treatment increases SIRT3 protein expression in GBM cells during ferroptosis

**Fig. 3:**
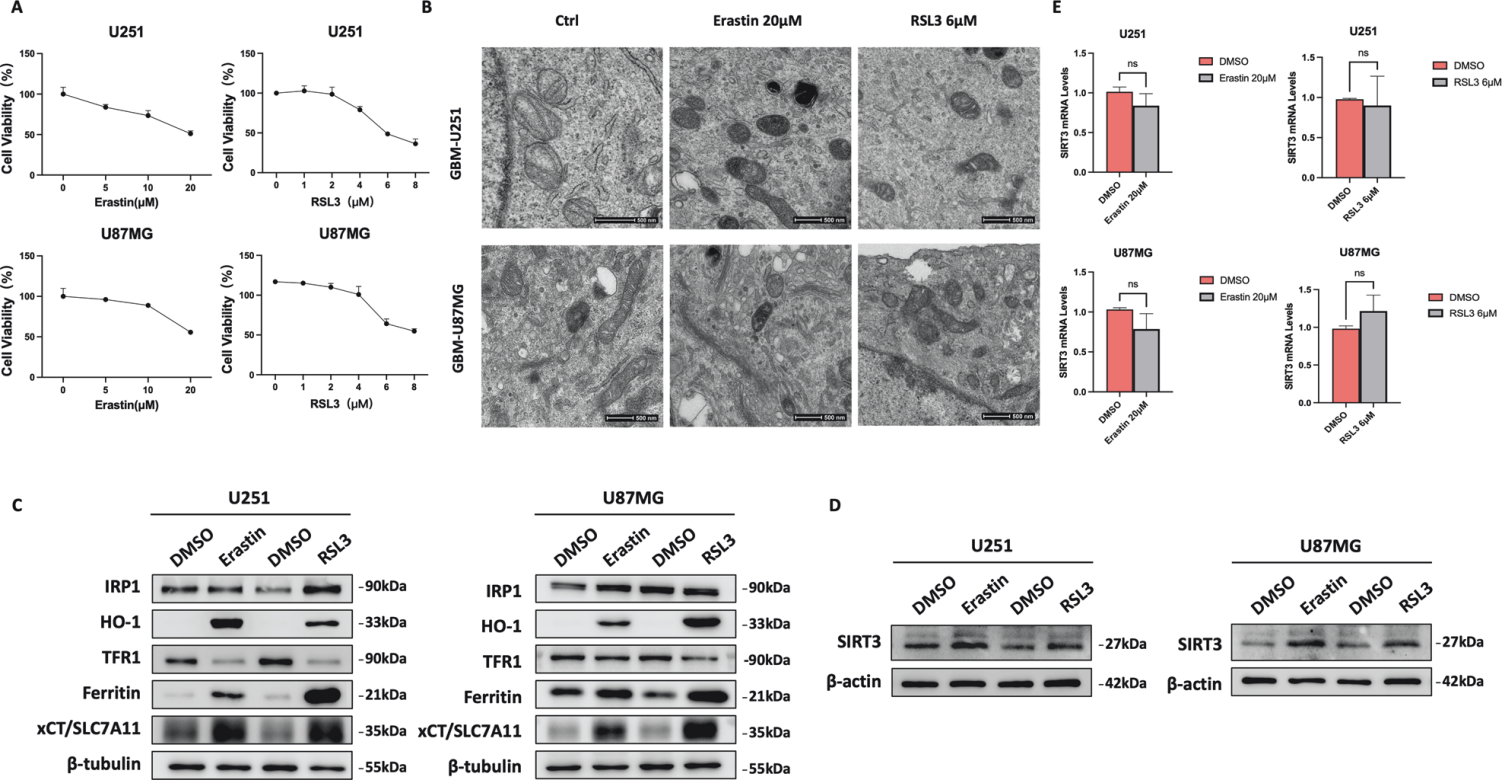
Erastin and RSL3 treatment led to increased SIRT3 protein expression during GBM cell ferroptosis. **A** Erastin and RSL3 treatment induced GBM cell death in a dose-dependent manner. **B** Erastin and RSL3 treatment induced typical ferroptosis-related morphology changes in mitochondria, as shown by transmission electron microscopy in GBM cells, including smaller mitochondria, higher density of the mitochondrial membrane, and reduced mitochondrial cristae numbers. **C** Erastin and RSL3 treatment increased expression of ferroptosis-related proteins in GBM cells. **D** Erastin and RSL3 treatment increased SIRT3 protein expression. **E** Erastin and RSL3 treatment did not affect *SIRT3* mRNA expression. IRP1, iron regulatory protein 1; HO-1, heme oxygenase 1; TFR1, transferrin 1; SLC7A11, solute family 7 member 11.

To investigate the involvement of SIRT3 in GBM cell ferroptosis, we treated GBM cell lines U251 and U87MG with ferroptosis inducers (FINs) erastin and RSL3. CCK8 assay results showed that erastin and RSL3 induced U251 and U87MG cell death in a dose-dependent manner (Fig. 3A). We then treated GBM cells with 20 μM erastin or 6 μM RSL3, individually, and observed changes in the morphology of the GBM cells upon FIN treatment by transmission electron microscopy. Both erastin and RSL3 reduced mitochondria size, increased mitochondrial membrane density, and diminished mitochondrial cristae (Fig. 3B). Moreover, erastin as well as RSL3 treatment increased the expression of a set of ferroptosis-related proteins, including iron regulatory protein 1 (IRP1), heme oxygenase 1 (HO-1), transferrin receptor 1 (TFR1), ferritin, and solute family 7 member 11 (SLC7A11), which collectively confirms the ferroptosis-inducing effects of erastin and RSL3 in GBM cells. During the induced ferroptosis of GBM cells by erastin and RSL3, SIRT3 expression was found to be upregulated at protein level but not at the mRNA level (Fig. 3D, E). We further found that treatment with DFO, an iron chelator, could not reverse the upregulation of SIRT3 protein upon RSL3 treatment (Fig. S4). However, treatment with NAC, a cystine analog as well as a deoxidizer, could reverse the upregulated SIRT3 expression (Fig. S4), indicating that the cellular cysteine level and, hence, oxidative stress might cause the increase in SIRT3 protein expression during FIN treatment. These data collectively demonstrate that SIRT3 is upregulated in induced GBM cell ferroptosis.

### SIRT3 protects GBM from RSL3-induced ferroptosis in vitro

**Fig. 4:**
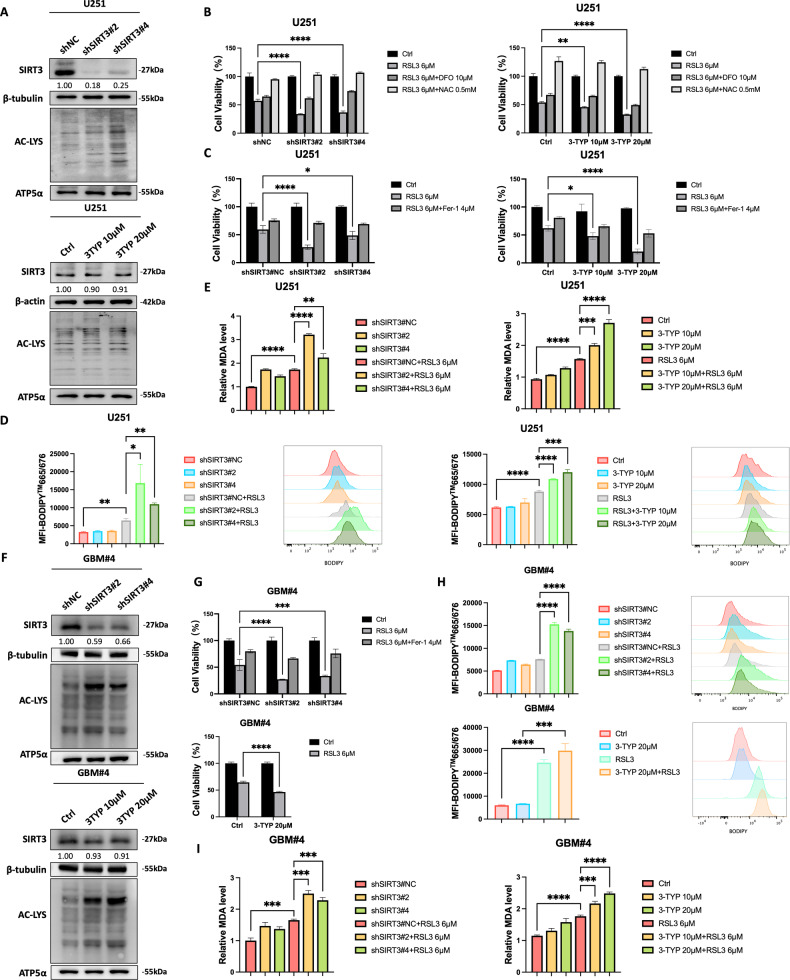
Inhibition of SIRT3 promoted RSL3-induced U251 and GBM4# cell ferroptosis in vitro. **A** Inhibition of SIRT3 expression and activity in U251 cells. **B** Inhibition of SIRT3 expression and activity promoted RSL3-induced U251 cell death, which was partially rescued by DFO and totally rescued by NAC in comparison with control cells. **C** Inhibition of SIRT3 expression and activity promoted RSL3-induced U251 cell death, which was partially rescued by Fer-1. **D** Inhibition of SIRT3 expression and activity promoted LPO accumulation in U251 cells. **E** Inhibition of SIRT3 expression and activity promoted MDA accumulation in U251 cells. **F** Inhibition of SIRT3 expression and activity in GBM#4 cells. **G** Inhibition of SIRT3 expression and activity promoted RSL3-induced GBM#4 cell death. **H** Inhibition of SIRT3 expression and activity promoted LPO accumulation in U251 cells. **I** Inhibition of SIRT3 expression and activity promoted MDA accumulation in U251 cells. *p < 0.05, **p < 0.01, ****p < 0.0001.

We isolated GBM#4 cells from primary surgical GBM biopsy specimens collected at Renji Hospital Affiliated to Shanghai Jiao Tong University School of Medicine. To determine whether high expression of SIRT3 protects GBM cells from ferroptosis, we inhibited SIRT3 in both genetic and chemical ways, by using a shRNA of *SIRT3* and the SIRT3 deacetylase activity specific inhibitor 3-TYP, individually. In the GBM cell line U251 and GBM#4 cells, reductions in SIRT3 expression and activity, respectively, upon treatment with shRNA and 3-TYP, individually, were confirmed by Western blotting analysis of the pan-acetylation level of mitochondrial proteins (Fig. 4A, F). When SIRT3 expression and activity were inhibited, RSL3 treatment induced more GBM cell death, which was partially rescued by application of DFO and totally rescued upon treatment with NAC in comparison with control cells (Fig. 4B,G). In U87MG cells, we observed the same enhanced cell death upon shRNA-mediated knock-down of SIRT3 (Fig. S5). We added ferrostatin-1 (Fer-1) treatment in the context of SIRT3 knock-down as well as 3-TYP treatment of U251 cells, and found that the GBM cell death promoted by SIRT3 inhibition could be partially rescued by fer-1, as shown in Fig. 4C. Moreover, targeting SIRT3 promoted more LPO and MDA accumulation in U251 and GBM4# cells (Fig. 4D, E, H, I), providing evidence that SIRT3 antagonizes GBM cell ferroptosis.

### SIRT3 inhibition combined with RSL3 treatment induces ferroptosis and attenuates GBM growth in vivo

**Fig. 5:**
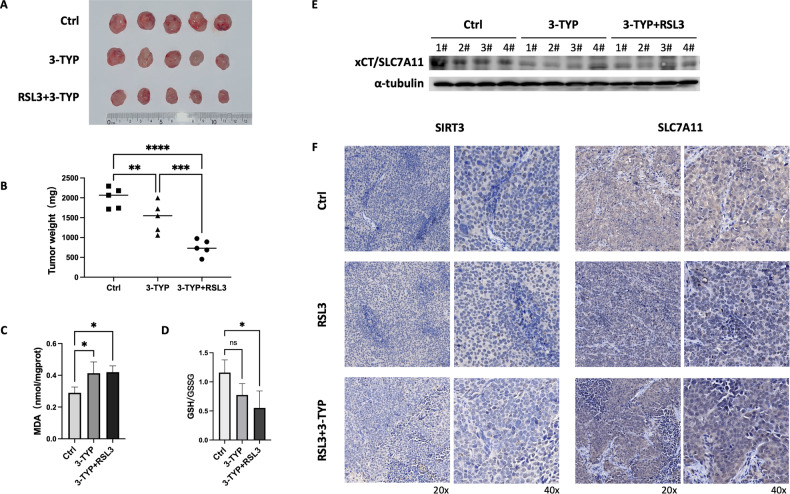
Inhibition of SIRT3 promoted RSL3-induced U251 cell ferroptosis in vivo. **A** 3-TYP + RSL3 treatments impeded GBM growth in xenograft mice, in comparison with the control and 3-TYP groups. **B** Quantitative results for tumor weights between the control, 3-TYP and 3-TYP + RSL3 treated groups. **C** MDA level was increased in 3-TYP and 3-TYP + RSL3 treated groups. **D** GSH/GSSG ratio was decreased in 3-TYP and 3-TYP + RSL3 treated groups. **E** Western blotting showed that 3-TYP treatment reduced SLC7A11 expression, while RSL3 treatment induced an adaptive increase in SLC7A11 in GBM tumor tissues in xenograft mice. **F** Immunohistochemical results showed that 3-TYP treatment had no effect on SIRT3 expression, but decreased SLC7A11 expression in tumor tissues in xenograft mice. Combination 3-TYP + RSL3 treatment increased both SIRT3 and SLC7A11 expression compared with 3-TYP treatment only.*p < 0.05, **p < 0.01, ***p < 0.001, ****p < 0.0001.

To further demonstrate the antagonizing role of SIRT3 against GBM ferroptosis in vivo, we tested the tumor inhibition effects of targeting SIRT3 before inducing ferroptosis. As shown in Fig. 5A,B, SIRT3 inhibition by 3-TYP in mice slowed GBM growth in vivo. Moreover, with combined treatment with 3-TYP and the ferroptosis inducer RSL3, tumor growth in vivo was more significantly suppressed. Furthermore, we detected that single inhibition of SIRT3 or combination with RSL3 treatment increased the MDA level while decreasing the GSH/GSSG ratio in tumor tissues, revealing that *S*IRT3 inhibition in combination with RSL3 treatment can attenuate GBM growth by inducing ferroptosis through a reduction in the GSH level in vivo (Fig. 5C,D). Indeed, we further found that 3-TYP treatment reduced SLC7A11 expression in tumor tissues, which was the reason for the decreased GSH/GSSG ratio in GBM tumors in xenograft mice (Fig. 5E, F). As shown in Fig. 5E, F, although RSL3 treatment caused an adaptive activation of the SIRT3-SLC7A11 axis, ferroptosis was still induced in vivo and hence resulted in significant slowing of GBM tumor growth.

### Targeting SIRT3 increases mitochondrial ROS level and Fe^2+^ concentration in GBM

**Fig. 6:**
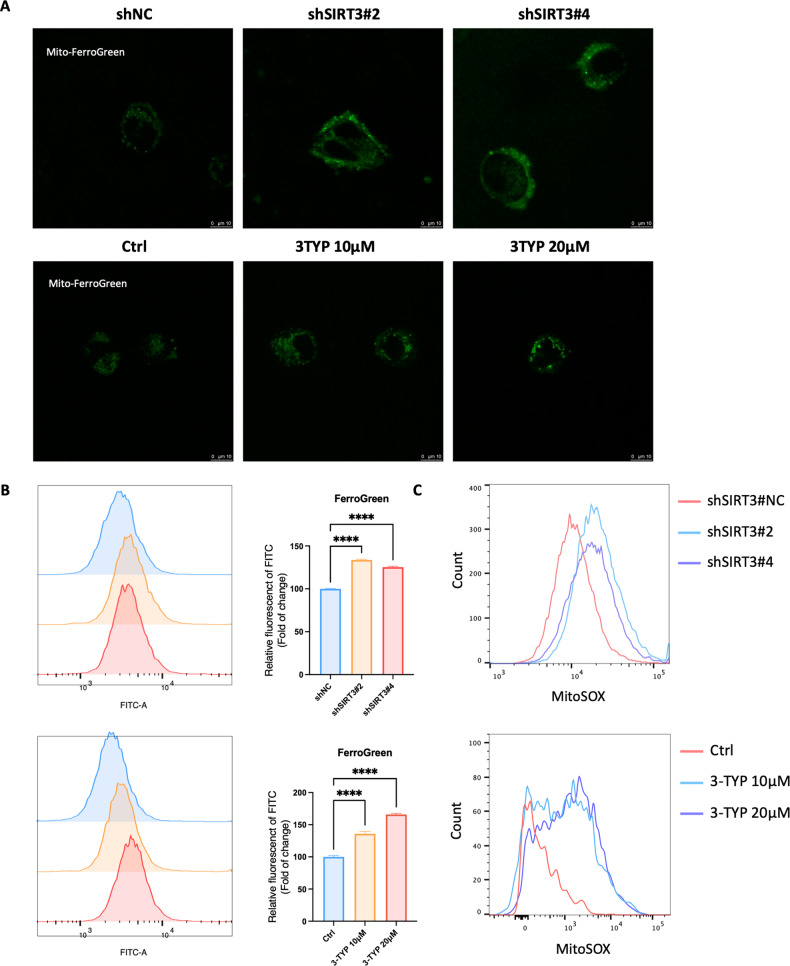
Inhibition of SIRT3 induced mitochondrial ROS and Fe^2+^ accumulation in U251 cells. **A** Mitochondrial Fe^2+^ level increased after SIRT3 inhibition. **B** FACS analysis of Mito-FerroGreen and quantitative results after SIRT3 inhibition. **C** Mitochondrial ROS level increased after SIRT3 inhibition.

Ferroptosis is a type of cell death promoted by accumulation of iron and ROS. Because SIRT3 is a mitochondrial protein with reported functions in iron and ROS level regulation [16], we next measured the mitochondrial Fe^2+^ and ROS levels in U251 cells upon SIRT3 inhibition. Mito-FerroGreen staining and MitoSOX assay revealed that under the condition of SIRT3 genetic or chemical inhibition, mitochondrial Fe^2+^ and ROS levels were both increased compared with those in control cells, illustrating the protective role of SIRT3 in GBM cell ferroptosis (Fig. 6). Besides, we performed FACS assays of Mito-FerroGreen, and the quantified results are presented in Fig. 6B.

### Targeting SIRT3 promotes mitophagy in GBM cells

**Fig. 7:**
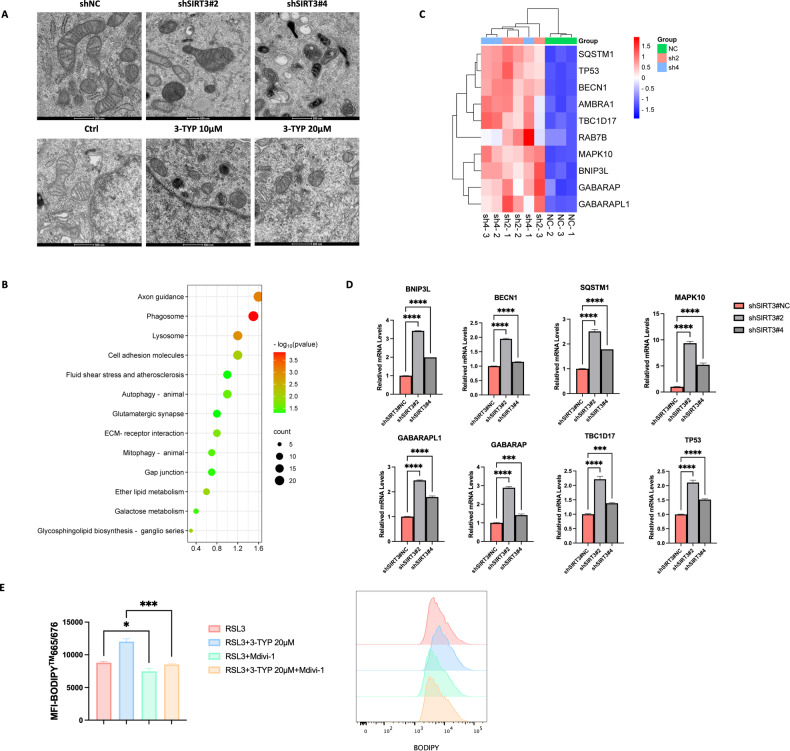
Mitophagy was increased in U251 cells upon SIRT3 knockdown. **A** Inhibition of SIRT3 induced mitophagy in U251 cells, as shown by transmission electron microscopy. **B** RNA-Seq revealed that mitophagy pathway genes were upregulated in U251 cells upon SIRT3 knockdown. **C** Heat map showing altered expression of genes in the mitophagy pathway. **D** Quantitative real-time PCR validation of altered expression of mitophagy-related genes in U251 cells upon SIRT3 knockdown. **E** Blocking mitophagy alleviated SIRT3-promoted, RSL3-induced U251 cell ferroptosis. ***p < 0.001, ****p < 0.0001.

Mitophagy has a reported role in priming cancer cells for ferroptosis [17, 18]. Due to the critical role of SIRT3 in regulating mitochondria quality and function, we investigated whether targeting SIRT3 could trigger mitophagy in GBM cells. As shown in Fig. 7A, inhibition of SIRT3 expression or activity significantly enhanced mitophagy among U251 cells (Fig. 7A). RNA-Seq analysis further revealed that mitophagy pathway genes were upregulated upon SIRT3 knockdown in GBM cells (Fig. 7B, C). Quantitative real-time PCR demonstrated the inhibitory role of SIRT3 on mitophagy-related gene expression in GBM cells (Fig. 7D), suggesting that targeting of SIRT3 sensitized GBM cells to ferroptosis at least partially by priming mitophagy.

Targeting SIRT3 downregulates SLC7A11 expression and cystine uptake in GBM cells

**Fig. 8:**
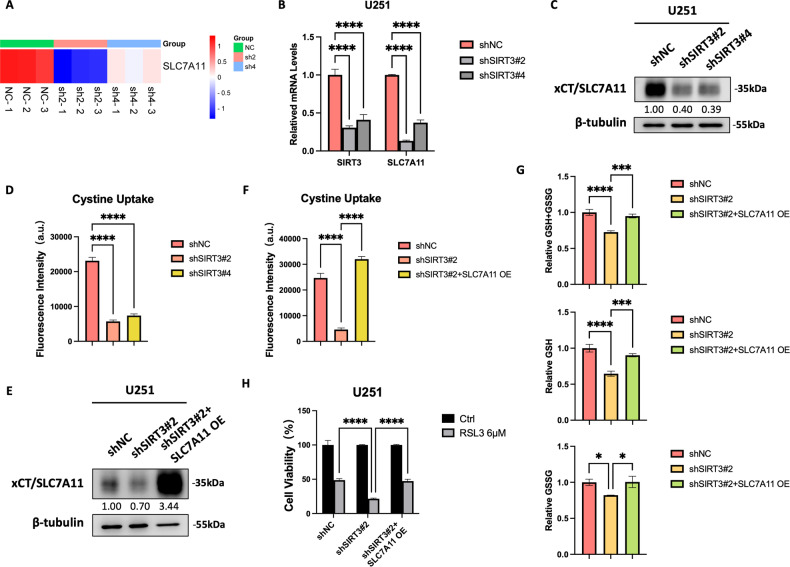
SIRT3 protected GBM cells from RSL3-induced ferroptosis partially through SLC7A11. **A** RNA-Seq showed that targeting of SIRT3 inhibited *SLC7A11* expression. **B** Quantitative real-time PCR also demonstrated that targeting of SIRT3 inhibited *SLC7A11* expression at the mRNA level. **C** Targeting of SIRT3 also inhibited SLC7A11 expression at the protein level. **D** Targeting of SIRT3 inhibited cystine uptake of GBM cells. **E** Forced expression of SLC7A11 in U251 cells with SIRT3 knockdown. **F** Forced expression of SLC7A11 in U251 cells with SIRT3 knockdown restored cystine uptake. **G** Forced expression of SLC7A11 in U251 cells with SIRT3 knockdown restored the cellular GSH level. **H** Forced expression of SLC7A11 in U251 cells with SIRT3 knockdown partially recovered cell viability following RSL3 treatment. *p < 0.05, ***p < 0.001, ****p < 0.0001.

SLC7A11 is crucial for ferroptosis antagonism and has been shown to be highly expressed in cancers and reflective of a poor prognosis [19]. Our results presented above identified a highly positive correlation between SIRT3 and SLC7A11 expression in GBM tissues (Fig. 2C). Furthermore, we detected that SIRT3 inhibition significantly decreased SLC7A11 expression in U251 cells upon RSL3 treatment (Fig. S6) and without RSL3 treatment (Fig. 8A–C). Targeting of SIRT3 in GBM cells also led to an obvious decrease in cystine uptake (Fig. 8D), and forced expression of SLC7A11 recovered cystine uptake in U251 cells with SIRT3 knockdown (Fig. 8E, F). Furthermore, by recovering cystine uptake, forced expression of SLC7A11 also rescued the GSH level that had been reduced by SIRT3 inhibition in U251 cells (Fig. 8G). However, we found that forced expression of SLC7A11 could only partially rescue the cell death promoted by SIRT3 inhibition, indicating that other mechanisms exist for targeting SIRT3 to sensitize GBM cells to RSL3-induced ferroptosis, in addition to the downregulation of SLC7A11 expression. Ferrous iron and LPO accumulation in mitochondria (Fig. 6) as well as enhanced mitophagy likely explain the ferroptosis promotion that occurs upon SIRT3 inhibition (Fig. 7).

### SIRT3 regulates SLC7A11 expression through ATF4 in GBM

**Fig. 9:**
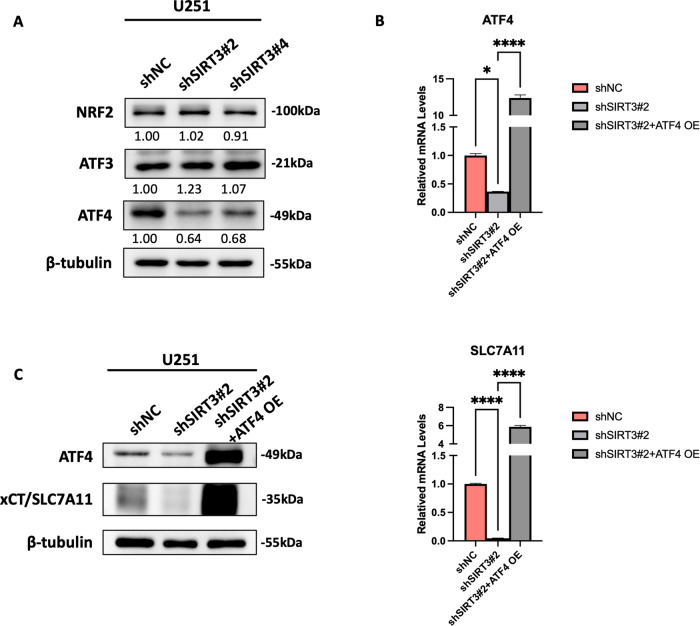
SIRT3 regulated SLC7A11 expression through ATF4. **A** Targeting of SIRT3 reduced ATF4 expression but not NRF2 and ATF3 expression. **B** Forced expression of ATF4 in U251 cells with SIRT3 knockdown rescued SLC7A11 expression at the mRNA level. **C** Forced expression of ATF4 in U251 cells with SIRT3 knockdown rescued SLC7A11 expression at the protein level. *p < 0.05, ****p < 0.0001.

Transcription factors, including NRF2, ATF3 and ATF4, mediate stress-induced *SLC7A11* transcription [19]. To explore the mechanism by which SIRT3 regulation affects *SLC7A11* expression, we first examined NRF2, ATF3 and ATF4 expression in U251 cells with SIRT3 knockdown. The results in Fig. 9A show that targeting SIRT3 dramatically reduced ATF4 expression, while no changes were observed in NRF2 and ATF3 expression in U251 cells, suggesting that SIRT3 specifically regulates ATF4 expression in GBM cells. We also detected reduced ATF4 expression at the mRNA level (Fig. 9B), illustrating that SIRT3 regulates ATF4 expression at the transcription level in GBM cells. We further restored ATF4 expression in U251 cells with SIRT3 knockdown and found that SLC7A11 expression was recovered (Fig. 9C). In addition, a previous study reported that SIRT3 regulates iron metabolism by modulating IRP1 activity [16]. The IRPs (IRP1 and IRP2) control the expression of iron-related genes by binding to the iron-responsive elements (IREs) of target mRNAs [20]. In the present study, we found that SIRT3 inhibition in GBM cells dramatically reduced IRP1 expression, while leaving no effects on IRP2 expression (Fig. S7A and S7B). Furthermore, we found that forced expression of IRP1 recovered the reduced expression of SLC7A11 caused by SIRT3 inhibition (Fig. S7C and S7D), indicating the role of IPR1 in the regulation of SLC7A11 expression in GBM cells, in the context of SIRT3 inhibition.

## Discussion

GBM is the most lethal type of primary brain tumor, because the lack of effective therapeutic methods leaves patients with a poor prognosis [21]. Therefore, research to discover novel treatment strategies and drugs are urgently needed. A very recent study by Watson et al. revealed that mitochondria are transferred from astrocytes to GBM cells where they reprogram the metabolism of GBM cells to enhance cell proliferation and tumorigenicity [22], indicating the promoting role of mitochondria in GBM progression. In the present study, we found that mitochondrial deacetylase SIRT3 protected GBM cells from RSL3-induced ferroptosis by inhibiting mitophagy and promoting SLC7A11 expression through ATF4 and IRP1. Targeted reduction of SIRT3 expression led to the accumulation of ferrous irons and increased lipid peroxidation in mitochondria, and ultimately ferroptosis. Therefore, targeting of SIRT3 in combination with ferroptosis induction may open a new avenue for conquering GBM.

The role of SIRT3 in various tumor types remains controversial [12]. The tumor suppressor or oncogene action of SIRT3 may depend on the tumor type and context [13, 23–26]. A recent study reported that fraxinellone, a tetrahydro-benzofuranone derivative, suppresses the growth and migration of GBM cells by downregulating SIRT3 signaling in vitro and inhibits the tumorigenesis of GBM in vivo [27]. In the present study, for the first time, we demonstrated the higher expression of SIRT3 in GBM cells in comparison with normal brain tissues. Moreover, we showed that high expression of SIRT3 in GBM predicted poor prognosis, implying that SIRT3 might inhibit tumor cell death by acting as an oncogene in GBM (Fig. 1). We found that upon FIN-induced ferroptosis, SIRT3 protein was upregulated, which might be an adaptation mechanism to resist FIN-induced cell death. We used genetic and chemical inhibition strategies to disrupt SIRT3 expression and activity in GBM cells and obtained evidence that targeting SIRT3 actually promoted RSL3-induced ferroptosis in vitro and inhibited GBM tumorigenesis and growth in vivo (Figs. 4 and 5).

Current evidence for the role of mitochondria in ferroptosis is growing but controversial [28]. Basit et al. reported that mitochondrial complex I inhibition triggers a mitophagy-dependent ROS increase, leading to ferroptosis in melanoma cells [17]. Targeting the oncogene Myoferlin in pancreatic cancer cells triggers mitophagy and promotes ferroptosis [18], indicating the priming role of mitophagy in tumor cell ferroptosis. As an important regulator on mitochondrial function and integrity, SIRT3 has been reported to play a mediatory role in mitophagy [29, 30]. The results of the present study showed that ferrous iron and ROS levels increased in mitochondria upon inhibition of SIRT3 expression and activity in GBM cells (Fig. 6), which could lead to destruction of mitochondrial integrity and trigger mitophagy, thereby promoting RSL3-induced ferroptosis.

SLC7A11 is well established as a key ferroptosis controller through modulation of the activity of the GSH-GPX4 antioxidant axis, the chief defense system during ferroptosis [19, 31–33]. The RNA-binding protein NKAP was reported to protect GBM cells from ferroptosis by promoting SLC7A11 mRNA splicing [6]. Our results showed that SIRT3 expression was highly correlated with SLC7A11 expression in GBM tissues (Fig. 2) and regulated SLC7A11 transcription in GBM cells (Fig. 8), including during the process of RSL3-induced ferroptosis (Fig. S6). The FIN II (ferroptosis inducer II) compound RSL3 targeted GPX4 to induce ferroptosis. We detected upregulation of both SIRT3 and SLC7A11 during RSL3-induced GBM cell ferroptosis (Fig. 3C,D). We consider that this SIRT3-regulated SLC7A11 compensation is induced by RSL3 as shown in Fig. 5E, F, which serves to increase the cellular GSH level, very likely due to the GPX4 activity inhibition by RSL3. However, how SIRT3 regulates *SLC7A11* transcription is worthy of investigation. Among the transcription factors reported to regulate SLC7A11 expression [19, 34–36], ATF4 is the only one found to be downregulated upon SIRT3 inhibition. Reduced ATF4 translation was previously observed in SIRT3-deficient diffuse large B-cell lymphoma cells [37]. However, in the present study, we found that targeted reduction of SIRT3 led to downregulation of ATF4 expression at the mRNA level, whereas rescued expression of ATF4 in SIRT3-deficient GBM cells restored SLC7A11 expression significantly (Fig. 9). In addition, SIRT3 was found to regulate cellular iron metabolism by modulating IRP1 activity [16]. In GBM cells, we further revealed that targeting SIRT3 inhibited IRP1 expression. However, the mechanism by which SIRT3 regulates IRP1 expression at the mRNA level, as well as the precise mechanism by which IRP1 regulates SLC7A11 expression, as an RNA-binding protein, need to be investigated further. Moreover, given SIRT3 is a deacetylase mainly located in mitochondria that greatly affects mitochondrial metabolism, we consider that SIRT3 may regulate SLC7A11 expression in an epigenetic manner. By affecting the abundance of metabolites in the TCA cycle, such as α-KG and succinate, SIRT3 regulates demethylase activity, the histone methylation level, and *SLC7A11* expression in GBM cells, and this needs to be verified in future work.

**Fig. 10:**
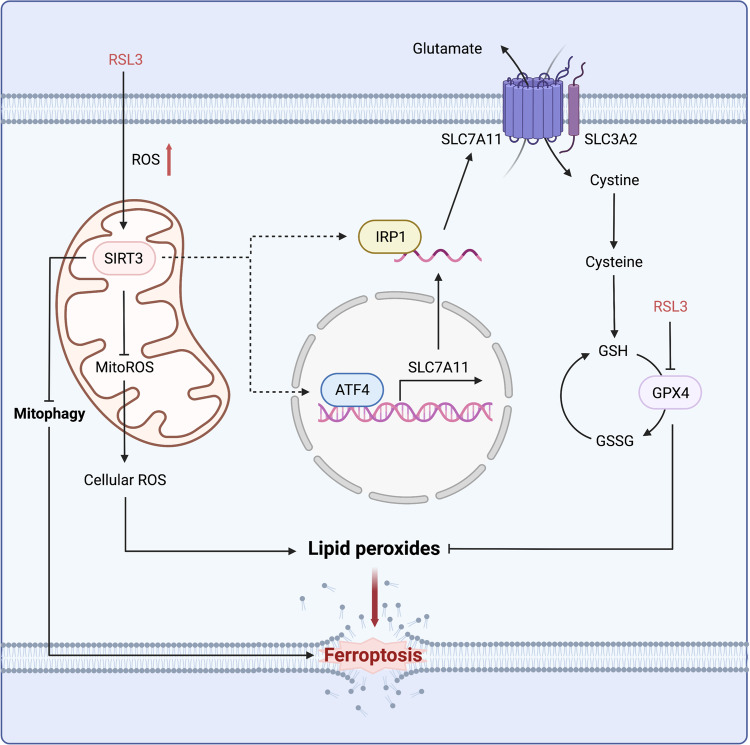
Working model depicting how targeted reduction of SIRT3 expression sensitizes GBM cells to RSL3-induced ferroptosis and the underlying mechanisms. SIRT3 is upregulated during RSL3-induced GBM cell ferroptosis, which protects GBM cells from ferroptosis by inhibiting mitophagy, and increasing SLC7A11 expression through ATF4 and IRP-1.

Increasing evidence for the role of SIRT3 in ferroptosis has emerged. SIRT3 deficiency in trophoblasts makes the cells resistant to autophagy-dependent ferroptosis by inhibiting the AMPK-mTOR pathway [38]. Liu et al. reported that SIRT3 inhibits bladder cancer progression by inducing AKT-dependent ferroptosis [14]. In contrast, in our study, we found that knockdown of SIRT3 did not alter AKT and AMPK-mTOR pathway activity in GBM cells (Fig. S8), indicating that SIRT3 regulates ferroptosis in a cell type- and context-dependent manner. Very recently, SIRT3 stabilization by deubiquitinase USP11 was found to significantly ameliorate oxidative stress-induced ferroptosis in intervertebral disc degeneration [39], suggesting an important role of SIRT3 in mitigating ferroptosis during disease. The results of the present study collectively demonstrated that SIRT3 inhibition markedly sensitized GBM cells to RSL3-induced ferroptosis, which in turn inhibited GBM growth and tumorigenesis. Because we did not distinguish IDH mutant tumors from IDH wild-type GBM in the human samples, whether SIRT3 has a differential regulatory effect on ferroptosis in IDH wild-type and IDH mutant GBM cells remains to be determined. However, because the cell lines and samples used in this study were all IDH wild-type, the current data support the finding that SIRT3 protects cells from ferroptosis in wild-type GBM. Our study further revealed that SIRT3 inhibition sensitized GBM cells to RSL3-induced ferroptosis by promoting mitophagy and reducing SLC7A11 expression (Fig. 10), providing insight into a potential combinatorial approach of targeting SIRT3 and inducing ferroptosis for GBM treatment.

## Conclusions

In summary, this study elucidates novel mechanisms underlying the ability of SIRT3 to protect GBM from ferroptosis. Because SIRT3 was more highly expressed in GBM samples than in normal brain tissue, we hypothesize that targeting SIRT3 to destroy mitochondrial integrity and homeostasis can damage tumor cells while leaving normal cells healthy. Thus, the combined strategy of inducing ferroptosis with a small molecule compound, such as RSL3, while also inhibiting SIRT3 represents a novel direction for GBM therapy.

### Supplementary information

Supplemental materials

Supplemental materials-final

## Data Availability

The authors confirm that the data supporting the findings of this study are available within the article and its supplementary materials.
