# Supplementary material for: Targeting SIRT3 sensitizes glioblastoma to ferroptosis by promoting mitophagy and inhibiting SLC7A11
Source: Cell Death Dis. 2024 Feb 23;15(2):168. doi: 10.1038/s41419-024-06558-0 (PMC10891132; doi:10.1038/s41419-024-06558-0)
Supplement: Supplementary file 2 — Supplemental materials-final [file 41419_2024_6558_MOESM2_ESM.docx]

**Supplemental materials**

*Correlation analysis between SIRT3 and ferroptosis-related genes (FRGs)*

Correlation analysis between SIRT3 expression and ferroptosis-related gene expression was performed using the RNA-Seq data for GBM and normal tissues from TCGA database and the Genotype-Tissue Expression (GTEx) database by GEPIA (http://gepia.cancer-pku.cn/).

*AKT and AMPK-mTOR pathway activity upon SIRT3 inhibition*

Phospho-AKT(Thr308), phospho-AKT(Ser473), AKT, phospho-AMPKα(Thr172), AMPKα, phospho-mTOR, and mTOR expression levels were detected by western blotting.

**Table S1 Antibodies and Reagents**

| **Reagents and Antibodies** | **Identifier** | | **Source** |
| --- | --- | --- | --- |
| ATF4 Rabbit Monoclonal Antibody | | AF2560 | Beyotime |
| ATF3 Rabbit Polyclonal Antibody | | AF6240 | Beyotime |
| p53 Mouse Monoclonal Antibody | | AF0255 | Beyotime |
| Beta Tubulin Rabbit Monoclonal Antibody | | AF1216 | Beyotime |
| Heme Oxygenase 1 (HO-1) Rabbit Monoclonal Antibody | | AF1333 | Beyotime |
| Anti-Ferritin Antibody | | ab75973 | Abcam |
| Anti-Nrf2 Antibody | | ab62352 | Abcam |
| Anti-SLC40A1 Antibody ab78066 | | ab78066 | Abcam |
| IRP2 Antibody (7H6) | | sc-33682 | Santa Cruz Biotechnology |
| Acetylated-Lysine Antibody | | 9441S | Cell Signaling Technology |
| Anti-SIRT3 Antibody | | 5490S | Cell Signaling Technology |
| IRP1 (D6S4J) Rabbit mAb | | 20272 | Cell Signaling Technology |
| xCT/SLC7A11 (D2M7A) Rabbit mAb | | 12691 | Cell Signaling Technology |
| Transferrin Receptor Antibody | | 13-6890 | Invitrogen |
| SLC7A11 Polyclonal Antibody | | PA1-16893 | Invitrogen |
| SIRT3 Polyclonal Antibody | | PA5-86035 | Invitrogen |
| Beta Actin Mouse Monoclonal Antibody | | 60008-1-Ig | Proteintech |
| Minimum Essential Medium (MEM) | | 11095 | Gibco |
| DH5α Competent Cell | | C502-03 | Vazyme |
| Mito-FerroGreen | | M489 | DOJINDO |
| MDA Assay Kit | | M496 | DOJINDO |
| Iron Assay Kit | | I291 | DOJINDO |
| FerroOrange | | F374 | DOJINDO |
| Cell Counting Kit-8 | | CK04 | DOJINDO |
| Cystine uptake assay kit | | UP05 | DOJINDO |
| NAC | | S0077 | Beyotime |
| GSH and GSSG Assay Kit | | S0053 | Beyotime |
| Reactive Oxygen Species Assay Kit | | S0033S | Beyotime |
| Iron Colorimetric Assay Kit | | K390 | Biovision |
| MitoSOX Red | | M36008 | Invitrogen |
| BODIPY 665/676 (Lipid Peroxidation Sensor) | | B3932 | Invitrogen |
| Erastin | | S7242 | Selleck |
| RSL3 | | S8155 | Selleck |
| 3-TYP | | S8628 | Selleck |
| Deferoxamine mesylate | | S5742 | Selleck |
| Glioblastoma Tissue Chip | | NCT805 | Taibsbio |
| Protein Removal Reagent Solution | | S0053-5 | Beyotime |

**Table S2 Details of samples in tissue microarray**

| No. | Age | Sex | Pathology diagnosis | Grade | Tissue ID. | Type |
| --- | --- | --- | --- | --- | --- | --- |
| 1 | 46 | F | Glioblastoma | 4 | Nct090105 | Malignant |
| 2 | 46 | F | Glioblastoma | 4 | Nct090105 | Malignant |
| 3 | 22 | F | Glioblastoma | 4 | Nct020020 | Malignant |
| 4 | 22 | F | Glioblastoma | 4 | Nct020020 | Malignant |
| 5 | 40 | M | Glioblastoma | 4 | Nct090045 | Malignant |
| 6 | 40 | M | Glioblastoma | 4 | Nct090045 | Malignant |
| 7 | 40 | F | Glioblastoma | 4 | Nct090029 | Malignant |
| 8 | 40 | F | Glioblastoma | 4 | Nct090029 | Malignant |
| 9 | 33 | F | Glioblastoma | 4 | Nct090002 | Malignant |
| 10 | 33 | F | Glioblastoma | 4 | Nct090002 | Malignant |
| 11 | 50 | F | Glioblastoma | 4 | Nct080110 | Malignant |
| 12 | 50 | F | Glioblastoma | 4 | Nct080110 | Malignant |
| 13 | 27 | F | High grade glioma | * | Nct080101 | Malignant |
| 14 | 27 | F | High grade glioma | * | Nct080101 | Malignant |
| 15 | 30 | F | Glioblastoma | 4 | Nct070128 | Malignant |
| 16 | 30 | F | Glioblastoma | 4 | Nct070128 | Malignant |
| 17 | 48 | F | Glioblastoma with necrosis | 4 | Nct060288 | Malignant |
| 18 | 48 | F | Glioblastoma with necrosis | 4 | Nct060288 | Malignant |
| 19 | 48 | F | Glioblastoma | 4 | Nct060389 | Malignant |
| 20 | 48 | F | Glioblastoma | 4 | Nct060389 | Malignant |
| 21 | 37 | F | Glioblastoma | 4 | Nct060303 | Malignant |
| 22 | 37 | F | Glioblastoma | 4 | Nct060303 | Malignant |
| 23 | 48 | F | Glioblastoma | 4 | Nct030071 | Malignant |
| 24 | 48 | F | Glioblastoma | 4 | Nct030071 | Malignant |
| 25 | 62 | M | Glioblastoma | 4 | Nct080070 | Malignant |
| 26 | 62 | M | Glioblastoma | 4 | Nct080070 | Malignant |
| 27 | 51 | M | Glioblastoma | 4 | Nct080080 | Malignant |
| 28 | 51 | M | Glioblastoma | 4 | Nct080080 | Malignant |
| 29 | 5 | M | Glioblastoma | 4 | Nct080027 | Malignant |
| 30 | 5 | M | Glioblastoma | 4 | Nct080027 | Malignant |
| 31 | 45 | M | High grade glioma | * | Nct080022 | Malignant |
| 32 | 45 | M | High grade glioma | * | Nct080022 | Malignant |
| 33 | 38 | F | Glioblastoma | 4 | Nct070179 | Malignant |
| 34 | 38 | F | Glioblastoma | 4 | Nct070179 | Malignant |
| 35 | 42 | F | Glioblastoma | 4 | Nct060275 | Malignant |
| 36 | 42 | F | Glioblastoma | 4 | Nct060275 | Malignant |
| 37 | 42 | M | Glioblastoma with degeneration | 4 | Nct060254 | Malignant |
| 38 | 42 | M | Glioblastoma with degeneration | 4 | Nct060254 | Malignant |
| 39 | 50 | F | Glioblastoma | 4 | Nct060160 | Malignant |
| 40 | 50 | F | Glioblastoma | 4 | Nct060160 | Malignant |
| 41 | 38 | M | Glioblastoma | 4 | Nct060123 | Malignant |
| 42 | 38 | M | Glioblastoma | 4 | Nct060123 | Malignant |
| 43 | 42 | M | Glioblastoma | 4 | Nct090016 | Malignant |
| 44 | 42 | M | Glioblastoma | 4 | Nct090016 | Malignant |
| 45 | 49 | M | Glioblastoma | 4 | Nct090026 | Malignant |
| 46 | 49 | M | Glioblastoma | 4 | Nct090026 | Malignant |
| 47 | 61 | M | Glioblastoma | 4 | Nct090085 | Malignant |
| 48 | 61 | M | Glioblastoma | 4 | Nct090085 | Malignant |
| 49 | 42 | M | Glioblastoma | 4 | Nct090090 | Malignant |
| 50 | 42 | M | Glioblastoma | 4 | Nct090090 | Malignant |
| 51 | 52 | F | Glioblastoma | 4 | Nct090118 | Malignant |
| 52 | 52 | F | Glioblastoma | 4 | Nct090118 | Malignant |
| 53 | 43 | M | Glioblastoma | 4 | Nct090017 | Malignant |
| 54 | 43 | M | Glioblastoma | 4 | Nct090017 | Malignant |
| 55 | 64 | M | Glioblastoma | 4 | Nct060370 | Malignant |
| 56 | 64 | M | Glioblastoma with necrosis | 4 | Nct060370 | Malignant |
| 57 | 36 | F | Glioblastoma | 4 | Nct090024 | Malignant |
| 58 | 36 | F | Glioblastoma | 4 | Nct090024 | Malignant |
| 59 | 65 | M | Glioblastoma with necrosis | 4 | Nct060390 | Malignant |
| 60 | 65 | M | Glioblastoma | 4 | Nct060390 | Malignant |
| 61 | 9 | M | Glioblastoma | 4 | Nct020175 | Malignant |
| 62 | 9 | M | Glioblastoma | 4 | Nct020175 | Malignant |
| 63 | 42 | F | Glioblastoma | 4 | Nct060373 | Malignant |
| 64 | 42 | F | Glioblastoma (sparse) | 4 | Nct060373 | Malignant |
| 65 | 25 | M | Glioblastoma | 4 | Nct040169 | Malignant |
| 66 | 25 | M | Glioblastoma | 4 | Nct040169 | Malignant |
| 67 | 66 | M | Glioblastoma | 4 | Nct050085 | Malignant |
| 68 | 66 | M | Glioblastoma | 4 | Nct050085 | Malignant |
| 69 | 67 | F | Glioblastoma | 4 | Nct070083 | Malignant |
| 70 | 67 | F | Glioblastoma | 4 | Nct070083 | Malignant |
| 71 | 15 | F | Cerebrum tissue | - | Nct06N002 | Normal |
| 72 | 15 | F | Cerebrum tissue | - | Nct06N002 | Normal |
| 73 | 35 | M | Cerebrum tissue | - | Nct05N006 | Normal |
| 74 | 35 | M | Cerebrum tissue | - | Nct05N006 | Normal |
| 75 | 48 | M | Cerebrum tissue | - | Nct05N023 | Normal |
| 76 | 48 | M | Cerebrum tissue | - | Nct05N023 | Normal |
| 77 | 42 | F | Cerebrum tissue | - | Nct03N006 | Normal |
| 78 | 42 | F | Cerebrum tissue | - | Nct03N006 | Normal |
| 79 | 21 | F | Cerebrum tissue | - | Nct11N001 | Normal |
| 80 | 21 | F | Cerebrum tissue | - | Nct11N001 | Normal |

**Table 3 Primers for qRT-PCR**

| Gene | Primer Sequence | |
| --- | --- | --- |
| 18s | Forward Sequence | GAAACGGCTACCACATCC |
|  | Reverse Sequence | CACCAGACTTGCCCTCCA |
| SIRT3 | Forward Sequence | CCCCAAGCCCTTTTTCACTTT |
|  | Reverse Sequence | CGACACTCTCTCAAGCCCA |
| BNIP3L | Forward Sequence | TGTGGAAATGCACACCAGCAGG |
|  | Reverse Sequence | CTACTGGACCAGTCTGATACCC |
| GABARAPL1 | Forward Sequence | TTGTAGAGAAGGCTCCAAAAGCC |
|  | Reverse Sequence | GGTCTCAGGTGGATTCTCTTCC |
| GABARAP | Forward Sequence | ATCTCCGAGCTGAGGATGCCTT |
|  | Reverse Sequence | GACACTTTCGTCACTGTAGGCAA |
| BECN1 | Forward Sequence | CTGGACACTCAGCTCAACGTCA |
|  | Reverse Sequence | CTCTAGTGCCAGCTCCTTTAGC |
| MAPK10 | Forward Sequence | GTGTGGAAGTGGGAGACTCAAC |
|  | Reverse Sequence | GTCAAGGACAGCATCATACGCG |
| SQSTM1 | Forward Sequence | TGTGTAGCGTCTGCGAGGGAAA |
|  | Reverse Sequence | AGTGTCCGTGTTTCACCTTCCG |
| TBC1D17 | Forward Sequence | TCCGCATGAAGCTGCAGTGGAA |
|  | Reverse Sequence | CCTCGTAGAACTTGTTGGTCCTG |
| TP53 | Forward Sequence | CCTCAGCATCTTATCCGAGTGG |
|  | Reverse Sequence | TGGATGGTGGTACAGTCAGAGC |
| SLC7A11 | Forward Sequence | TCTCCAAAGGAGGTTACCTGC |
|  | Reverse Sequence | AGACTCCCCTCAGTAAAGTGAC |
| IRP1 | Forward Sequence | AACCCATTCGCACACCTTG |
|  | Reverse Sequence | ATGGTAAGCGCCCATATCTTG |

**
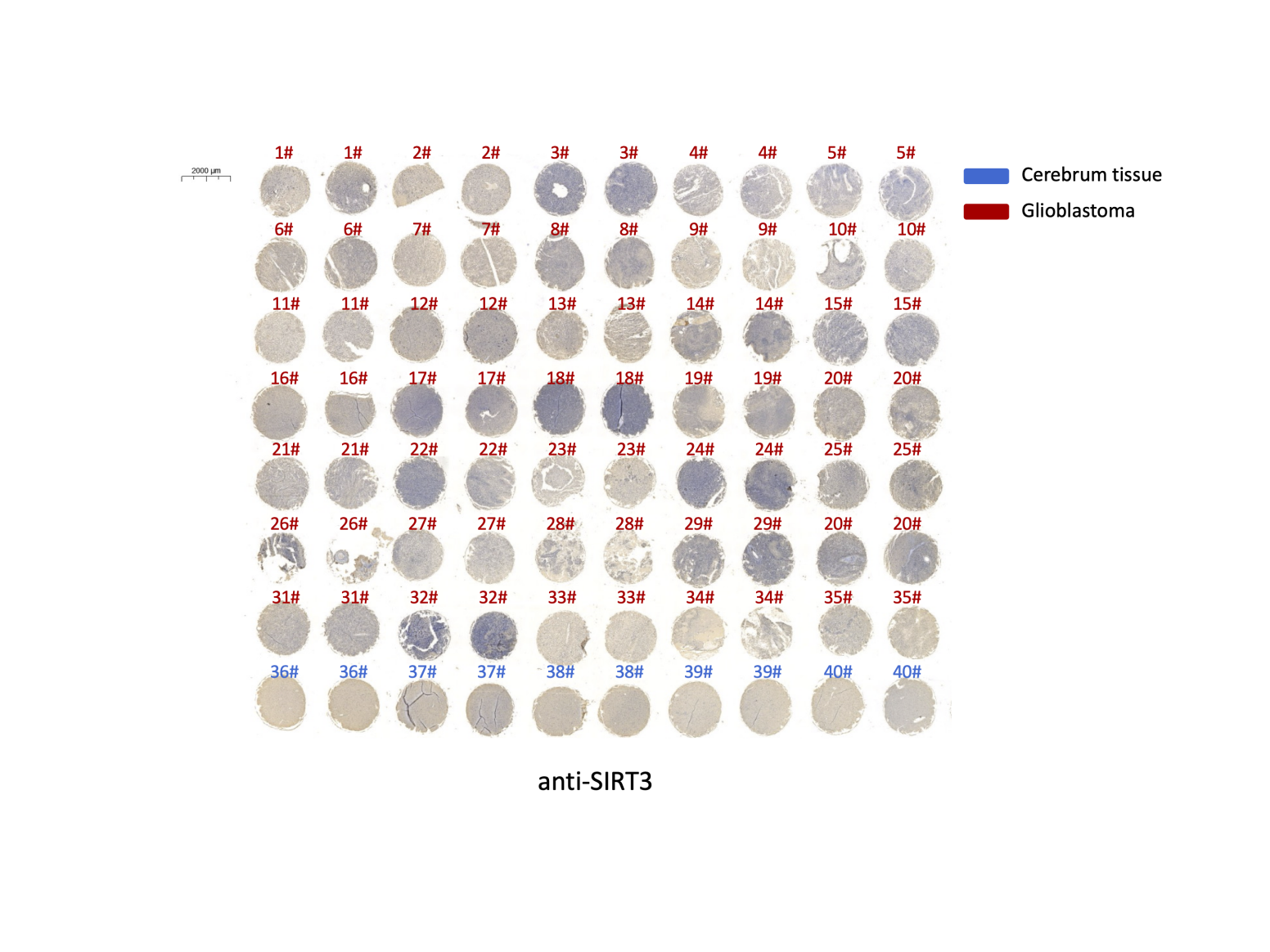
**

**Fig. S1** SIRT3 expression in tissue microarray.


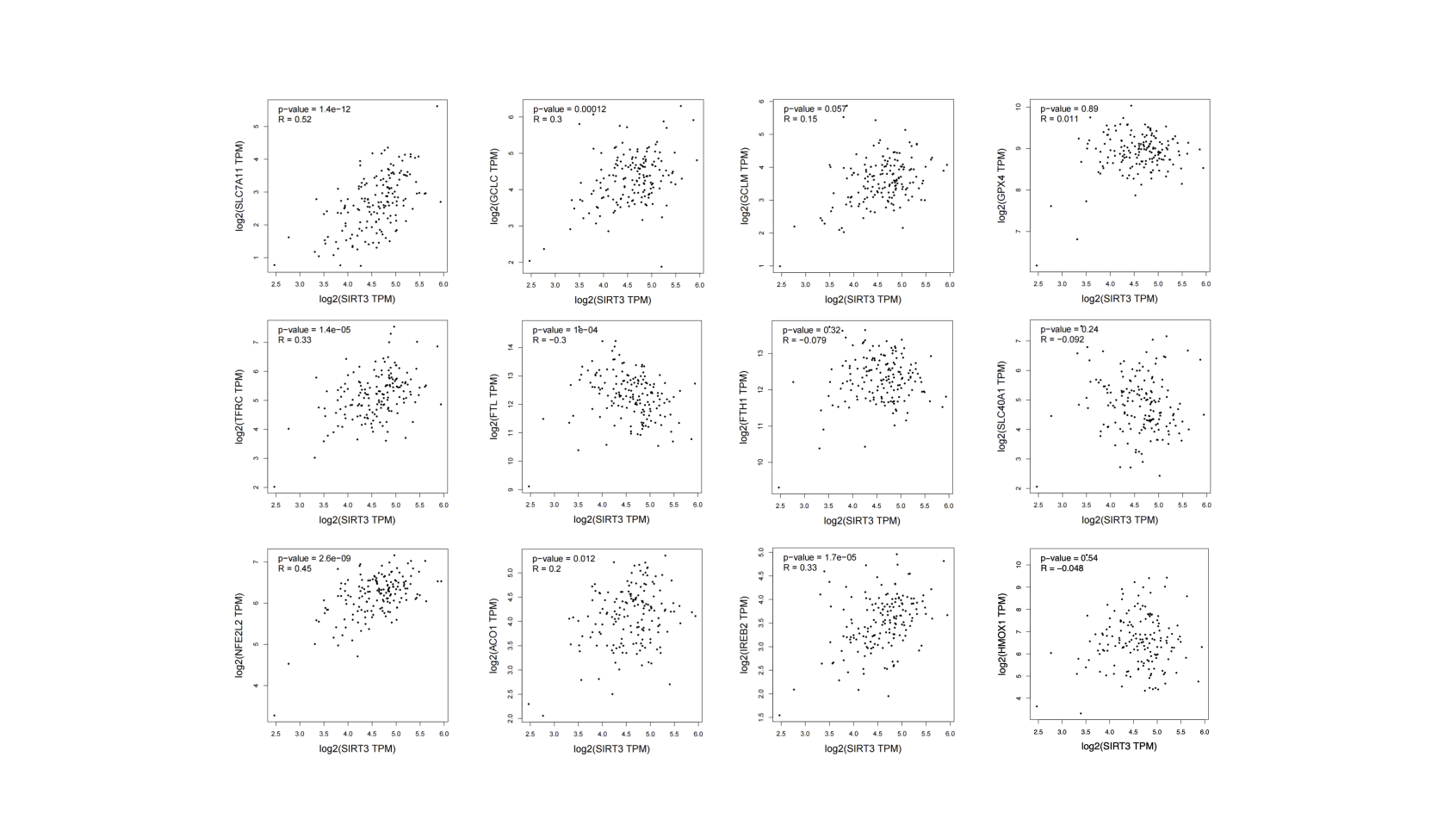


**Fig. S2** SIRT3 expression was positively correlated with SLC7A11 expression.


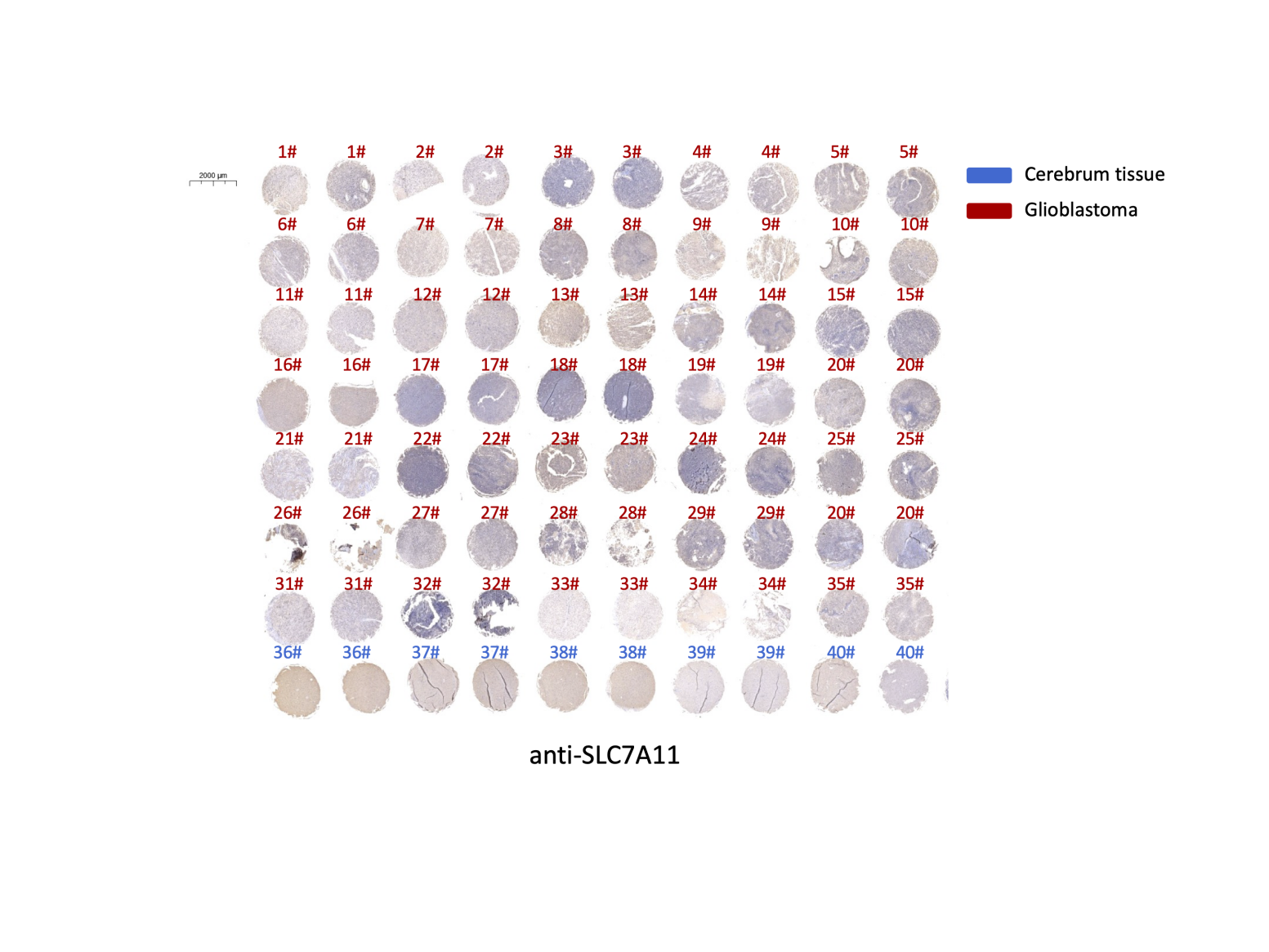


**Fig. S3** SLC7A11 expression in tissue microarray.


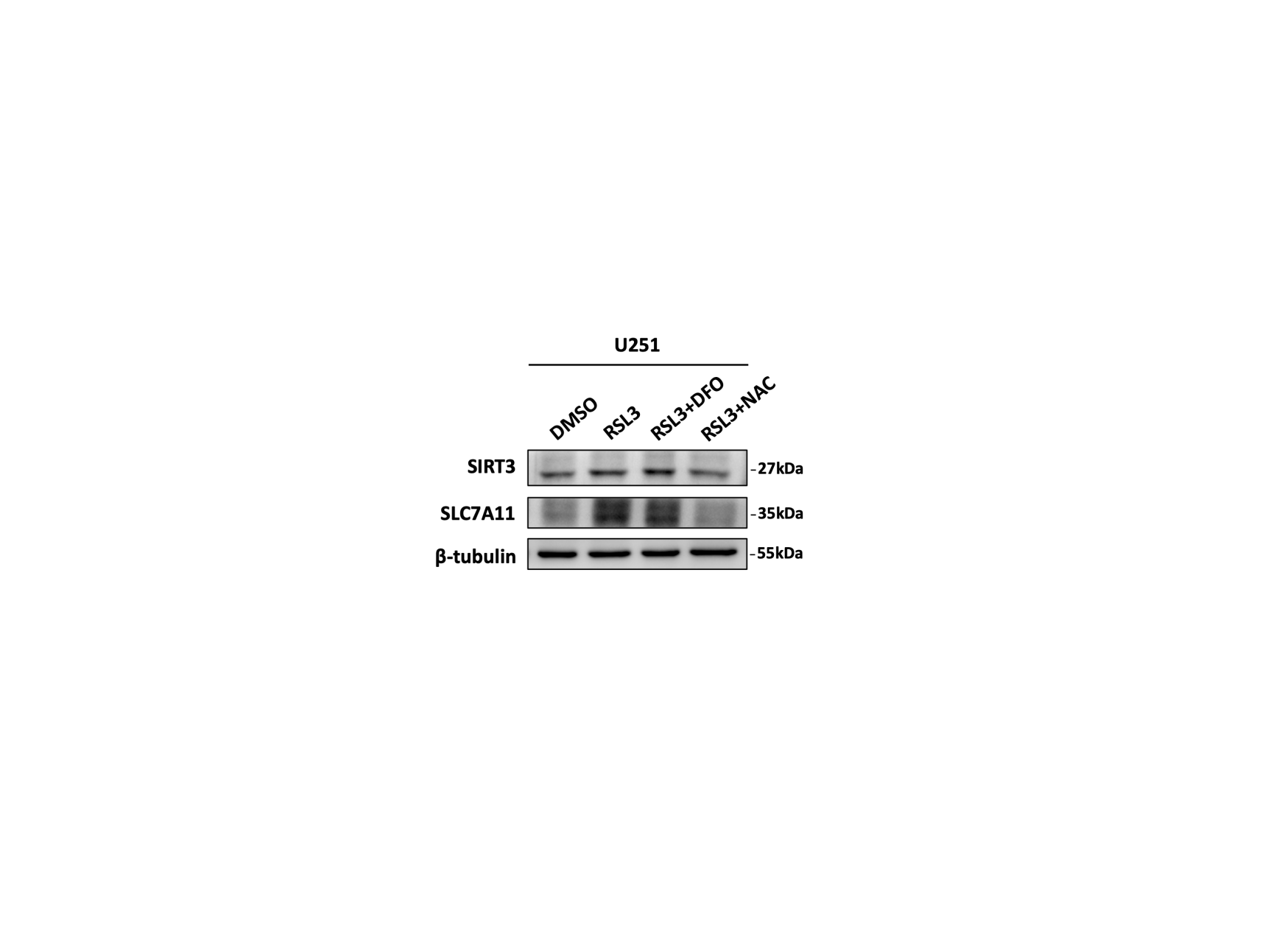


**Fig. S4** Treatment with NAC to reduce ROS level reversed RSL3-induced SIRT3 and SLC7A11 protein upregulation.


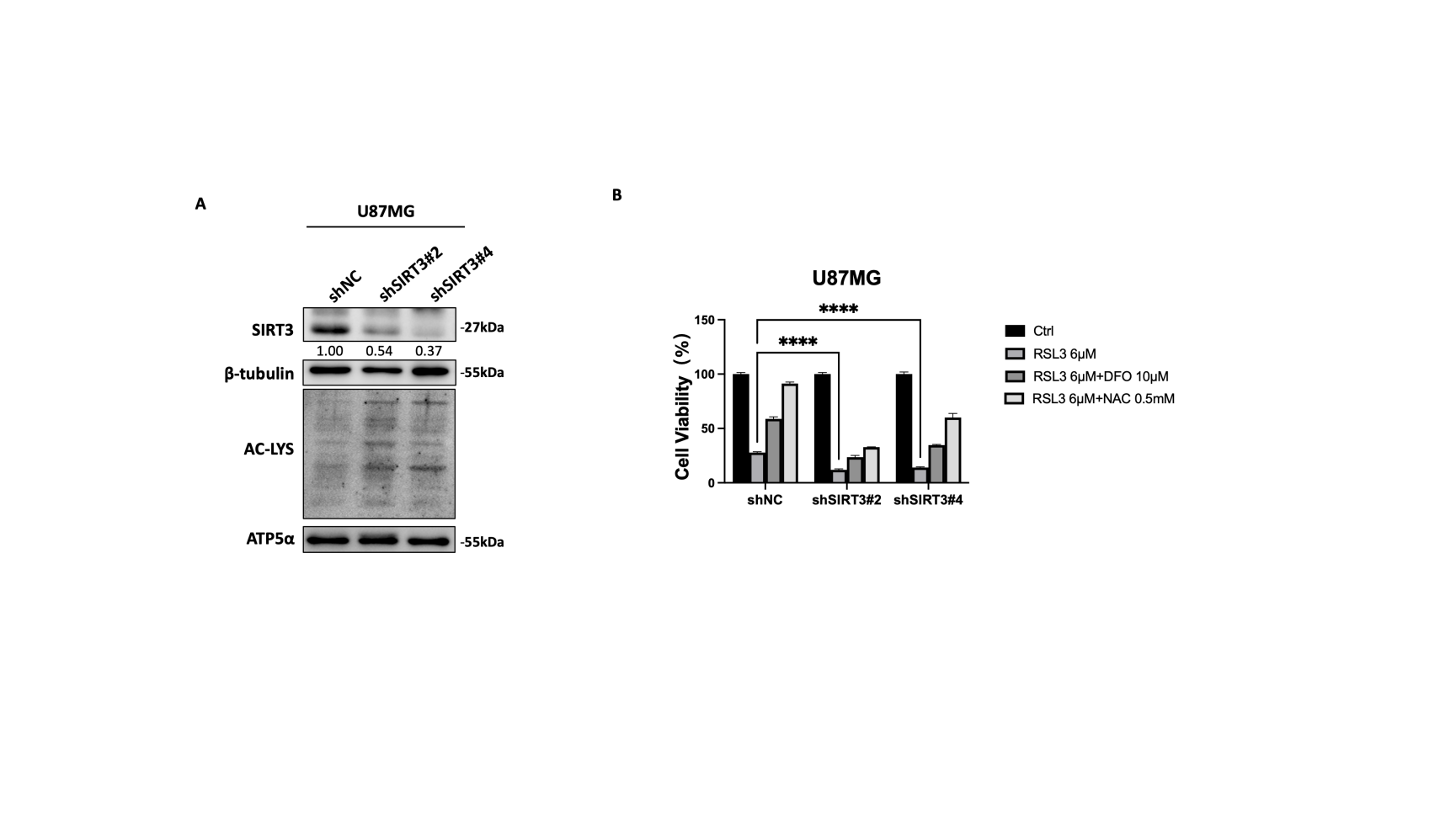


**Fig. S5** Inhibition of SIRT3 promoted RSL3-induced U87MG cell ferroptosis *in vitro.* **A** Knockdown of SIRT3 in U87MG cells upregulated pan-acetylation in mitochondria. **B** Inhibition of SIRT3 promoted RSL3-induced U87MG cell death, which was partially rescued by DFO and totally rescued by NAC in comparison with control cells. ****p<0.0001.


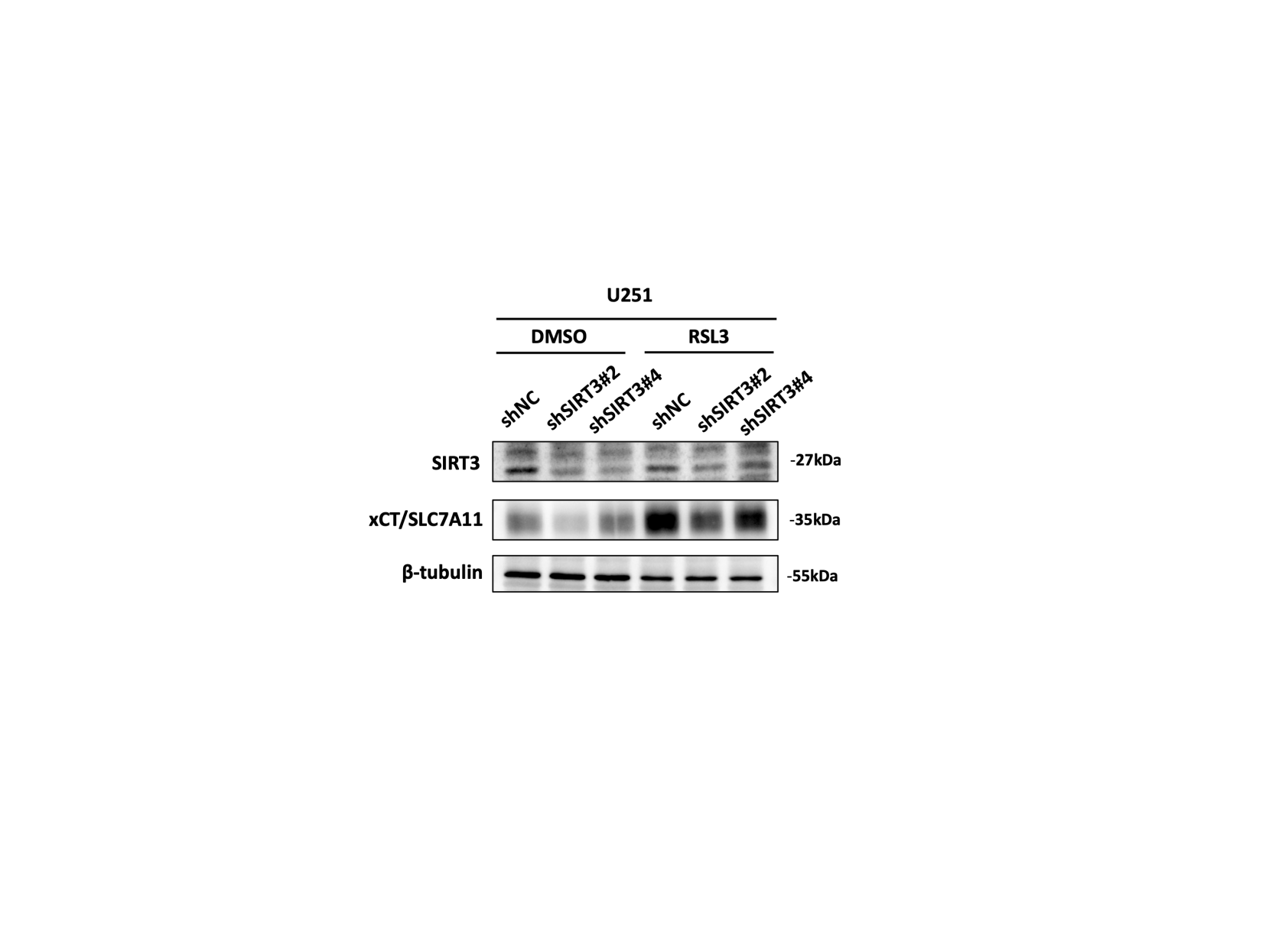


**Fig. S6** SIRT3 inhibition reduced SLC7A11 expression upon RSL3 treatment.


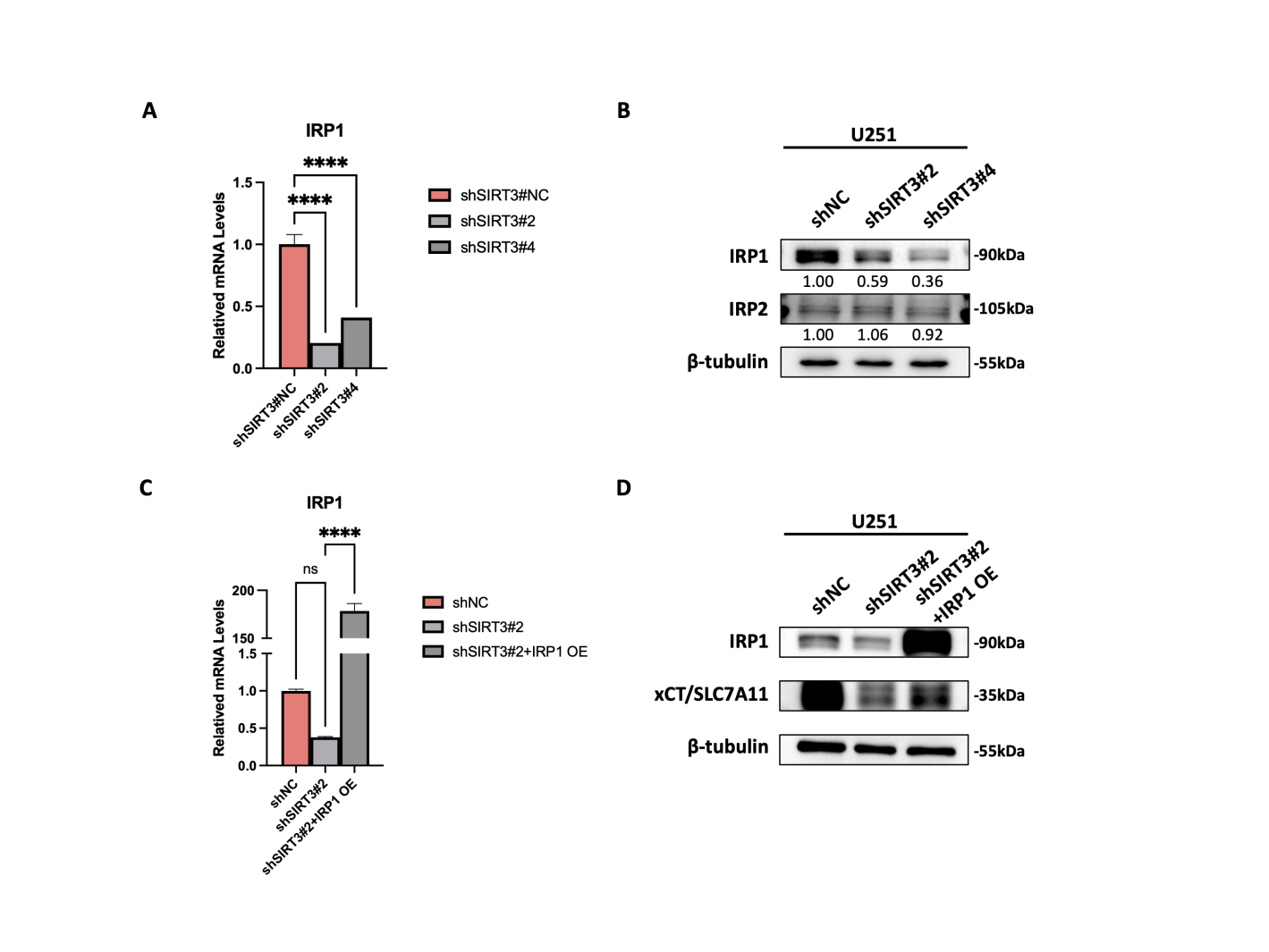


**Fig. S7** SIRT3 expression regulated SLC7A11 expression through IRP1. **A** Knockdown of SIRT3 inhibited IRP1 expression in U251 cells at mRNA level. **B** Knockdown of SIRT3 inhibited IRP1 expression in U251 cells at protein level. **C** Forced expression of IRP1 in U251 cells with SIRT3 knockdown rescued SLC7A11 expression at the mRNA level. **D** Forced expression of IRP1 in U251 cells with SIRT3 knockdown rescued SLC7A11 expression at the protein level. ****p<0.0001.


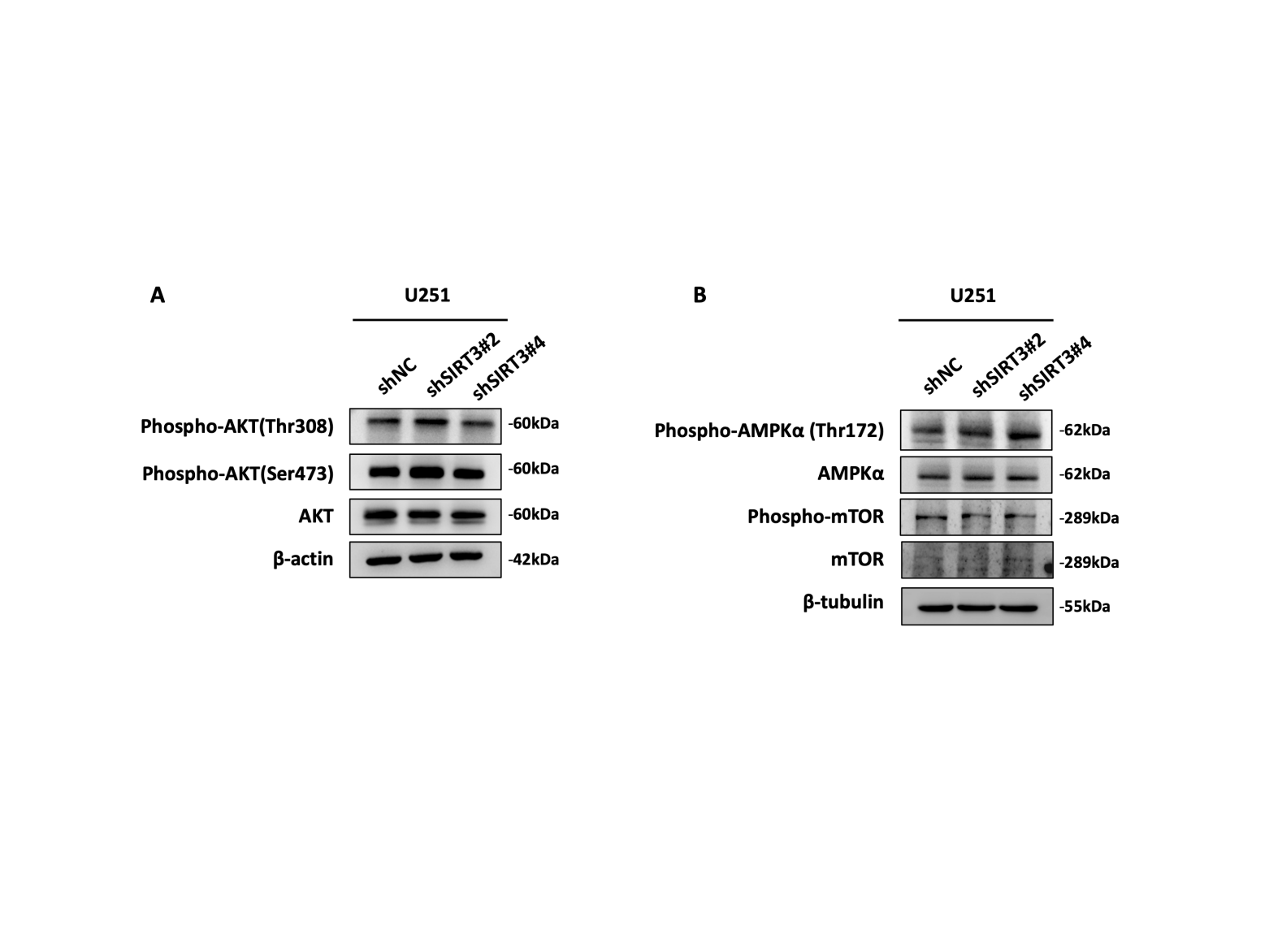


**Fig. S8** Knockdown of SIRT3 did not alter the signaling activities of the AKT and AMPK-mTOR pathways in U251 cells. **A** Knockdown of SIRT3 did not alter AKT pathway signaling activity. **B** Knockdown of SIRT3 did not alter AMPK-mTOR pathway signaling activity.
