# Supplementary material for: Targeting SIRT3 sensitizes glioblastoma to ferroptosis by promoting mitophagy and inhibiting SLC7A11
Source: Cell Death Dis. 2024 Feb 23;15(2):168. doi: 10.1038/s41419-024-06558-0 (PMC10891132; doi:10.1038/s41419-024-06558-0)
Supplement: Supplementary file 1 — Supplemental materials [file 41419_2024_6558_MOESM1_ESM.docx]

**HO-1**

**Ferritin**

**TFR1**

**IRP1**

**xCT/SLC7A11**

**β-tubulin**

**RSL3**

**DMSO**

**Erastin**

**DMSO**

**U251**

-**90kDa**

-**55kDa**

-**35kDa**

-**21kDa**

-**90kDa**

-**33kDa**

**HO-1**

**Ferritin**

**TFR1**

**IRP1**

**xCT/SLC7A11**

**β-tubulin**

**RSL3**

**DMSO**

**Erastin**

**DMSO**

**U87MG**

-**90kDa**

-**55kDa**

-**35kDa**

-**21kDa**

**-90kDa**

-**33kDa**

**Figure3 C**

**xCT/SLC7A11**

**HO-1**

**Ferritin**

**TFR1**

**IRP1**

**β-tubulin**

**RSL3**

**DMSO**

**Erastin**

**DMSO**

**U251**

-**90kDa**

-**55kDa**

-**35kDa**

-**21kDa**

-**90kDa**

-**33kDa**

**HO-1**

**Ferritin**

**TFR1**

**IRP1**

**xCT/SLC7A11**

**β-tubulin**

**RSL3**

**DMSO**

**Erastin**

**DMSO**

**U87MG**

-**90kDa**

-**55kDa**

-**35kDa**

-**21kDa**

-**90kDa**

-**33kDa**

**SIRT3**

**β-actin**

**RSL3**

**DMSO**

**Erastin**

**DMSO**

**U251**

**-27kDa**

**-42kDa**

**SIRT3**

**β-actin**

**RSL3**

**DMSO**

**Erastin**

**DMSO**

**U87MG**

**-27kDa**

**-42kDa**

**Figure3 D**

**SIRT3**

**β-actin**

**RSL3**

**DMSO**

**Erastin**

**DMSO**

**U87MG**

**-27kDa**

**-42kDa**

**SIRT3**

**β-actin**

**RSL3**

**DMSO**

**Erastin**

**DMSO**

**U251**

**-27kDa**

**-42kDa**

**SIRT3**

**shSIRT3#2**

**β-tubulin**

**shSIRT3#4**

**-27kDa**

**-55kDa**

**shNC**

**AC-LYS**

**ATP5α**

**-55kDa**

**1.00**

**0.18**

**0.25**

**U251**

**Figure4 A**

**SIRT3**

**shSIRT3#2**

**β-tubulin**

**shSIRT3#4**

**-27kDa**

**-55kDa**

**shNC**

**AC-LYS**

**ATP5α**

**-55kDa**

**U251**

**Ctrl**

**3TYP 10μM**

**3TYP 20μM**

**-27kDa**

**-42kDa**

**-55kDa**

**SIRT3**

**β-actin**

**AC-LYS**

**ATP5α**

**1.00**

**0.90**

**0.91**

**U251**

**Figure4 A**

**Ctrl**

**3TYP 10μM**

**3TYP 20μM**

**-27kDa**

**-42kDa**

**-55kDa**

**SIRT3**

**β-actin**

**AC-LYS**

**ATP5α**

**U251**

**Figure4 F**

**SIRT3**

**-27kDa**

**AC-LYS**

**ATP5α**

**-55kDa**

**GBM#4**

**shSIRT3#2**

**shSIRT3#4**

**shNC**

**β-tubulin**

**-55kDa**

**SIRT3**

**-27kDa**

**AC-LYS**

**ATP5α**

**-55kDa**

**GBM#4**

**shSIRT3#2**

**shNC**

**1.00**

**0.59**

**0.66**

**β-tubulin**

**-55kDa**

**shSIRT3#4**

**Figure4 F**

**Ctrl**

**3TYP 10μM**

**3TYP 20μM**

**SIRT3**

**-27kDa**

**AC-LYS**

**ATP5α**

**-55kDa**

**GBM#4**

**β-tubulin**

**-55kDa**

**Ctrl**

**3TYP 10μM**

**3TYP 20μM**

**SIRT3**

**-27kDa**

**AC-LYS**

**ATP5α**

**-55kDa**

**GBM#4**

**β-tubulin**

**-55kDa**

**1.00**

**0.93**

**0.91**

**Figure5 E**

**xCT/SLC7A11**

**Ctrl**

**α-tubulin**

**3-TYP**

**3-TYP+RSL3**

1#

2#

3#

4#

1#

2#

3#

4#

1#

2#

3#

4#

**xCT/SLC7A11**

**Ctrl**

**α-tubulin**

**3-TYP**

**3-TYP+RSL3**

1#

2#

3#

4#

1#

2#

3#

4#

1#

2#

3#

4#

-**35kDa**

-**55kDa**

**xCT/SLC7A11**

**U251**

**shSIRT3#2**

**β-tubulin**

**shSIRT3#2+**

**SLC7A11 OE**

**-35kDa**

**-55kDa**

**shNC**

**Figure8 C**

**xCT/SLC7A11**

**U251**

**shSIRT3#2**

**β-tubulin**

**shSIRT3#4**

**-35kDa**

**-55kDa**

**shNC**

**1.00**

**0.39**

**0.40**

**xCT/SLC7A11**

**U251**

**shSIRT3#2**

**β-tubulin**

**shSIRT3#2+**

**SLC7A11 OE**

**-35kDa**

**-55kDa**

**shNC**

**1.00**

**3.44**

**0.70**

**Figure8 E**

**xCT/SLC7A11**

**U251**

**shSIRT3#2**

**β-tubulin**

**shSIRT3#4**

**-35kDa**

**-55kDa**

**shNC**

**U251**

**shSIRT3#2**

**shSIRT3#4**

**β-tubulin**

**-55kDa**

**ATF4**

**-49kDa**

**ATF3**

**-21kDa**

**NRF2**

**-100kDa**

**shNC**

1.00

1.02

0.91

1.00

1.23

1.07

1.00

0.64

0.68

**Figure9 A**

**U251**

**shSIRT3#2**

**shSIRT3#4**

**β-tubulin**

**-55kDa**

**ATF4**

**-49kDa**

**ATF3**

**-21kDa**

**NRF2**

**-100kDa**

**shNC**

**ATF4**

**β-tubulin**

**xCT/SLC7A11**

**U251**

**shSIRT3#2**

**shSIRT3#2**

**+ATF4 OE**

**shNC**

**-55kDa**

**-49kDa**

**-35kDa**

**Figure9 C**

**ATF4**

**β-tubulin**

**xCT/SLC7A11**

**U251**

**shSIRT3#2**

**shSIRT3#2**

**+ATF4 OE**

**shNC**

**-55kDa**

**-49kDa**

**-35kDa**

**FigureS4**

SIRT3

β-tubulin

**RSL3+NAC**

**DMSO**

**RSL3**

**RSL3+DFO**

**U251**

-**27kDa**

-**55kDa**

SLC7A11

-**35kDa**

SIRT3

β-tubulin

**RSL3+NAC**

**DMSO**

**RSL3**

**RSL3+DFO**

**U251**

-**27kDa**

-**55kDa**

SLC7A11

-**35kDa**

**U87MG**

**β-tubulin**

**SIRT3**

**shSIRT3#2**

**shSIRT3#4**

**shNC**

**AC-LYS**

**ATP5α**

**-27kDa**

**-55kDa**

**-55kDa**

**1.00**

**0.54**

**0.37**

**FigureS5 A**

**U87MG**

**β-tubulin**

**SIRT3**

**shSIRT3#2**

**shSIRT3#4**

**shNC**

**AC-LYS**

**ATP5α**

**-27kDa**

**-55kDa**

**-55kDa**

**FigureS6**

**xCT/SLC7A11**

**β-tubulin**

**-35kDa**

**-55kDa**

**SIRT3**

**-27kDa**

**U251**

**shSIRT3#2**

**shSIRT3#4**

**shNC**

**shSIRT3#2**

**shSIRT3#4**

**shNC**

**DMSO**

**RSL3**

**xCT/SLC7A11**

**β-tubulin**

-**35kDa**

-**55kDa**

**SIRT3**

-**27kDa**

**U251**

**shSIRT3#2**

**shSIRT3#4**

**shNC**

**shSIRT3#2**

**shSIRT3#4**

**shNC**

**DMSO**

**RSL3**

**IRP1**

**U251**

**shSIRT3#2**

**IRP2**

**shSIRT3#4**

**β-tubulin**

**-105kDa**

**-55kDa**

**-90kDa**

**shNC**

**1.00**

**0.59**

**0.36**

**1.00**

**1.06**

**0.92**

**IRP1**

**β-tubulin**

**xCT/SLC7A11**

**U251**

**shSIRT3#2**

**shSIRT3#2**

**+IRP1 OE**

**shNC**

**-55kDa**

**-90kDa**

**-35kDa**

**FigureS7 B**

**FigureS7 D**

**IRP1**

**β-tubulin**

**xCT/SLC7A11**

**U251**

**shSIRT3#2**

**shSIRT3#2**

**+IRP1 OE**

**shNC**

**-55kDa**

**-90kDa**

**-35kDa**

**IRP1**

**U251**

**shSIRT3#2**

**IRP2**

**shSIRT3#4**

**β-tubulin**

**-105kDa**

**-55kDa**

**-90kDa**

**shNC**

**FigureS8 A**

**AKT**

**Phospho-AKT(Ser473)**

**Phospho-AKT(Thr308)**

-**60kDa**

-**60kDa**

-**60kDa**

**β-actin**

-**42kDa**

**U251**

**shSIRT3#2**

**shSIRT3#4**

**shNC**

**AKT**

**Phospho-AKT(Ser473)**

**Phospho-AKT(Thr308)**

-**60kDa**

-**60kDa**

-**60kDa**

**β-actin**

-**42kDa**

**U251**

**shSIRT3#2**

**shSIRT3#4**

**shNC**

**mTOR**

**Phospho-mTOR**

**AMPKα**

**Phospho-AMPKα (Thr172)**

**-62kDa**

**-62kDa**

**-289kDa**

**-289kDa**

**β-tubulin**

**-55kDa**

**U251**

**shSIRT3#2**

**shSIRT3#4**

**shNC**

**mTOR**

**Phospho-mTOR**

**AMPKα**

**Phospho-AMPKα (Thr172)**

**-62kDa**

**-62kDa**

**-289kDa**

**-289kDa**

**β-tubulin**

**-55kDa**

**U251**

**shSIRT3#2**

**shSIRT3#4**

**shNC**

**FigureS8 B**
